# The RNA helicase RHAU (DHX36) suppresses expression of the transcription factor PITX1

**DOI:** 10.1093/nar/gkt1340

**Published:** 2013-12-24

**Authors:** Evan P. Booy, Ryan Howard, Oksana Marushchak, Emmanuel O. Ariyo, Markus Meier, Stefanie K. Novakowski, Soumya R. Deo, Edis Dzananovic, Jörg Stetefeld, Sean A. McKenna

**Affiliations:** ^1^Department of Chemistry, University of Manitoba, Winnipeg, Manitoba, Canada, ^2^University of Manitoba, Winnipeg, Manitoba, Canada, ^3^Department of Biochemistry and Molecular Biology, University of British Columbia, V6T 1Z4 Vancouver, British Columbia, Canada and ^4^Department of Biochemistry and Medical Genetics, University of Manitoba, Winnipeg, Manitoba, Canada, R3T 2N2

## Abstract

RNA Helicase associated with AU-rich element (RHAU) (DHX36) is a DEAH (Aspartic acid, Glumatic Acid, Alanine, Histidine)-box RNA helicase that can bind and unwind G4-quadruplexes in DNA and RNA. To detect novel RNA targets of RHAU, we performed an RNA co-immunoprecipitation screen and identified the PITX1 messenger RNA (mRNA) as specifically and highly enriched. PITX1 is a homeobox transcription factor with roles in both development and cancer. Primary sequence analysis identified three probable quadruplexes within the 3′-untranslated region of the PITX1 mRNA. Each of these sequences, when isolated, forms stable quadruplex structures that interact with RHAU. We provide evidence that these quadruplexes exist in the endogenous mRNA; however, we discovered that RHAU is tethered to the mRNA via an alternative non–quadruplex-forming region. RHAU knockdown by small interfering RNA results in significant increases in PITX1 protein levels with only marginal changes in mRNA, suggesting a role for RHAU in translational regulation. Involvement of components of the microRNA machinery is supported by similar and non-additive increases in PITX1 protein expression on Dicer and combined RHAU/Dicer knockdown. We also demonstrate a requirement of argonaute-2, a key RNA-induced silencing complex component, to mediate RHAU-dependent changes in PITX1 protein levels. These results demonstrate a novel role for RHAU in microRNA-mediated translational regulation at a quadruplex-containing 3′-untranslated region.

## INTRODUCTION

Guanine-rich nucleic acids are prone to fold into unique structures known as a G4-quadruplexes. These quadruplexes typically consist of four tracts of guanines arranged in parallel or anti-parallel strands that align in stacked tetrad planes. These tetrad guanine planes are stabilized by Hoogsteen hydrogen bonds and a monovalent cation (1). Quadruplexes are present in both DNA and RNA and enable multifaceted control of gene expression and post-transcriptional gene regulation (2,3). Quadruplexes within gene promoters have recently been demonstrated to recruit transcription factors to regulate gene expression at a transcriptional level (4–6). Moreover, quadruplexes within the untranslated regions of mRNAs are implicated in translational regulation, splicing, polyadenylation and mRNA stability (3,7–9). The discovery and development of numerous quadruplex-stabilizing compounds suggests great potential for the targeting of these regulatory structures for therapeutic intervention (10,11).

**R**NA **H**elicase associated with **AU**-rich element (RHAU), also known as DHX36, is a member of the DEAH-box family of RNA helicases that has emerged as a key enzyme capable of binding and unwinding both inter- and intramolecular quadruplexes in both DNA and RNA (12–14). RHAU binds both DNA and RNA quadruplexes with high affinity and specificity via a conserved N-terminal region known as the RHAU-specific motif (RSM) (14,15). A minimal fragment of RHAU containing the RSM (RHAU_53__–__105_) is sufficient for specific quadruplex interaction; however, the full-length protein containing the helicase domain is necessary for the quadruplex unfolding activity of RHAU (12,14). Recent studies have demonstrated a quadruplex-dependent role for RHAU in the regulation of the *YY1* and *TNAP* genes, and we and others have shown that RHAU is a critical helicase in the remodelling of quadruplexes within the 5′-end of the human telomerase RNA (5,6,12,16,17). In addition to an initial study showing an impact of RHAU on mRNA stability through binding an AU-rich element in the 3′-untranslated region (UTR) of the urokinase plasminogen activator mRNA, RHAU is also implicated in microRNA (miRNA)-mediated gene regulation through both miRNA translocation and direct interactions with human argonaute proteins (18–21). To expand on the known functions of RHAU and identify novel quadruplex-containing RNAs, we carried out an RNA immunoprecipitation screen by pulling down endogenous RHAU–RNA complexes from human cell lysates followed by RNA isolation and identification. This approach yielded several candidate RNAs, one of which is the mRNA of the transcription factor PITX1.

PITX1, a homeobox transcription factor, plays a pivotal role in the differentiation of the developing pituitary gland (22). PITX1 also has important functions in limb development, as mutations in PITX1 are found in individuals with hereditary limb deformities (23–25). Although a role in development has been the primary focus of PITX1 studies, several recent reports suggest an additional role as a tumour suppressor gene. PITX1 expression is downregulated in a number of tumour types including lung, colorectal, gastric and esophageal cancer, and reduced PITX1 expression has been correlated with decreased overall patient survival (26–29). Furthering its role as a tumour suppressor, an RNA-interference library screen identified PITX1 as a potent inhibitor of Rat sarcoma-mitogen activated protein kinase (RAS-MAPK) signalling through transcriptional upregulation of *RASAL1* (30). Moreover, PITX1 expression is increased in response to DNA damage, and it acts as a direct transcriptional upregulator of p53, a critical component of the DNA damage response that is frequently mutated in many types of cancer (31,32). Adding to its role as a tumour suppressor gene is a recent study that identified PITX1 as a negative regulator of telomerase reverse transcriptase, an enzyme critical for the unlimited replication characteristic of tumour cells (33). As PITX1 possesses important functions in both development and disease, and because the mRNA exhibited high and specific affinity for RHAU, it was selected as a candidate for further study.

In the present report, we confirm a specific interaction between endogenously expressed RHAU and the PITX1 mRNA. Three regions within the PITX1 3′-UTR that are prone to fold into stable quadruplexes and appear to persist in the endogenous mRNA are identified. Reporter assays implicate the most 3′ quadruplex-forming region as involved in RHAU-dependent PITX1 regulation. We characterize a specific interaction between RHAU and the PITX1 3′-UTR and demonstrate that, although the isolated quadruplex-binding domain of RHAU can interact with the PITX1 mRNA, the full-length protein is tethered via an alternative protein–protein or protein–RNA interaction that is quadruplex-independent and occurs over a short region near the end of the 3′-UTR. An indirect interaction is particularly likely in that we demonstrate the helicase-dead RHAU mutant demonstrates similar binding to the RNA as the wild-type protein. Involvement of miRNA-mediated pathways is supported through small interfering RNA (siRNA) studies of Dicer that demonstrate similar and non-additive increases in PITX1 protein on Dicer and RHAU knockdown. Additionally, argonaute-2 (Ago2) knockdown is found to prevent RHAU siRNA-dependent upregulation of PITX1. This study, for the first time, identifies PITX1 as a quadruplex-containing RNA that is bound by RHAU at the 3′ UTR to regulate protein expression.

## MATERIALS AND METHODS

### Cell culture and reagents

The HEK293T cell line was a gift from Dr Thomas Klonisch, and the HeLa and Michigan Cancer Center Foundation-7 (MCF-7) cell lines were a gift from Dr Spencer Gibson. The monoclonal murine anti-RHAU antibody was a gift from Dr Yoshikuni Nagamine. Cell culture and monoclonal antibody purification was performed as described previously (12). The following additional antibodies were used: mouse anti-synthetic hapten (isotype control; Abcam, Toronto, ON, Canada), mouse anti-α-tubulin (T6074, Sigma-Aldrich, Oakville, ON, Canada), rabbit anti-pitx1, (ab72073, Abcam), mouse anti-Ago2 (ab57113, Abcam), rabbit anti-Ago2 (2897S, Cell Signaling Technology, Boston, MA, USA), rabbit anti-Dicer (5362S, Cell Signaling Technology) and anti-6×His Tag antibody (ab18184, Abcam). Synthetic RNAs and DNA primers were purchased from Integrated DNA Technologies (Coralville, IA, USA). All oligonucleotides were provided desalted and sequence confirmed by mass spectrometry. The quadruplex stain *N*-methyl-mesoporphyrin IX was purchased from Frontier Scientific (Logan, UT, USA). All standard laboratory chemicals and reagents were purchased from Thermo-Fisher Scientific (Ottawa, ON, Canada). The recombinant full-length RHAU protein and RHAU_53__–__105_ truncation were purified as described previously (12).

### RNA immunoprecipitation, cloning and sequencing

To screen for novel RHAU binding RNAs, cells from 20 confluent 150-mm dishes were scraped into 10 ml of cold phosphate-buffered saline per plate and pelleted by centrifugation at 1500*g* for 5 min at 4°C. The cell pellet was resuspended in 5 ml of cytoplasmic lysis buffer [25 mM Hepes, pH 7.9, 5 mM KCl, 0.5 mM MgCl_2_, 0.5% (v/v) NP-40 supplemented with protease and RNase inhibitors (Halt protease and phosphatase inhibitor cocktail and Riblock RNase inhibitor, Thermo-Fisher Scientific)]. The cells were lysed on ice for 5 min and centrifuged at 5000 rpm in a bench-top microfuge for 5 min at 4°C. The supernatant was set aside on ice, and the cell pellet was resuspended in 5 ml of nuclear lysis buffer (25 mM Hepes, pH 7.9, 10% (w/v) sucrose, 350 mM NaCl, 0.01% (v/v) NP-40 supplemented with protease/phosphatase/RNase inhibitors). To lyse the nuclei, the suspension was vortexed for 30 s followed by passage through a 20-gauge needle. The nuclear and cytoplasmic fractions were combined, and insoluble material was removed by centrifugation at 14 000rpm in a bench-top microfuge at 4°C for 10 min. A total of 150 µg of monoclonal anti-RHAU antibody was added to the cell lysate and incubated for 30 min at room temperature with end-over-end mixing. After 30 min, 500 µl of Pierce Protein A/G magnetic beads (Thermo-Fisher Scientific) pre-equilibrated in lysis buffer was added to the sample, and mixing was continued for 30 min at room temperature. The magnetic beads were subsequently washed 3-fold with cytoplasmic lysis buffer, followed by three washes with nuclear lysis buffer and finally three washes with a 1:1 mixture of cytoplasmic and nuclear lysis buffer.

To isolate the copurified RNA, beads were resuspended in 300 µl of resuspension buffer from the Thermo-Scientific GeneJet RNA cleanup and concentration micro kit, and the RNA was purified according to the manufacturer's instructions. Ribosomal RNA was removed from the preparation using the Ribo-Zero rRNA removal kit (Epicentre Biotechnologies, Madison, WI, USA). RNA was then reverse-transcribed to complementary DNA (cDNA) using the Revertaid H minus first strand cDNA synthesis kit according to the manufacturer’s instructions (Thermo-Fisher Scientific). Second strand synthesis was performed by combining 25 µl of the first strand synthesis mixture with 40 U *Escherichia coli* DNA Polymerase (New England Biolabs, Ipswich, MA, USA), 2 U RNase H (New England Biolabs), 300 µM dNTP mix (New England Biolabs) and 10 µl NE Buffer 2 (New England Biolabs). The reaction was brought to a final volume of 100 µl and incubated at 15°C for 2 h. After 2 h, 10 U of T4 DNA polymerase was added to the reaction and incubation continued for an additional 5 min. The double-stranded cDNA was then purified using the GeneJet PCR purification kit (Thermo-Fisher Scientific). Purified cDNA was blunt-end cloned into the pJet1.2 vector using the CloneJET PCR cloning kit (Thermo-Fisher Scientific) and transformed into chemically competent *E. coli*. Plasmid DNA was isolated from transformants using the GeneJet plasmid DNA mini-prep kit (Thermo-Fisher Scientific) and sequenced (Eurofins MWG Operon, Huntsville, Alabama, USA).

### RNA immunoprecipitation for RT-PCR analysis

For real-time PCR analysis, RNA was isolated and purified as aforementioned using a single confluent 150-mm dish. A total of 2 µl (∼20 ng) of the purified RNA was used directly with the iScript one-step reverse transcriptase–polymerase chain reaction (RT–PCR) kit (BioRad Laboratories, Hercules, CA USA) according to the manufacturer's protocol with an annealing temperature of 55°C. Fold enrichment values were calculated relative to 25 ng total RNA. The cDNA was amplified by quantitative PCR with primers specific to the RNA transcripts of PITX1, TERC, BCYRN1 and Glyceraldehyde 3-phosphate dehydrogenase (GAPDH). For RHAU_53__–__105_ competition assays, 5 µM RHAU_53__–__105_ was added to the binding reaction for 30 min before addition of antibody. For the 6×His-RHAU_53__–__105_ immunoprecipitations, the binding reaction was supplemented with 300 nM 6×His-RHAU_53__–__105_ for 30 min before addition of the anti-6×His Tag antibody. Cycling conditions were as follows: 95°C for 5 min followed by 40 cycles of 95°C for 10 s and 55°C for 30 s. The PCR products were analyzed by agarose gel electrophoresis following amplification to ensure reaction specificity. The primer sequences used are as follows: PITX1 coding sequence (CDS) forward—TCTCCACCAAGAGCTTCACCTTCT, PITX1 CDS reverse—GCCGGTGAGGTTGTTGATGTTGTT, PITX1 UTR forward—TTTACAGCGTCCCTGTGTATG, PITX1 UTR reverse—CACGCTCGGACTATGGTTT, hTR-forward—TCTAACCCTAACTGAGAAGGGCGT, hTR-reverse—TGCTCTAGAATGAACGGTGGAAGG, BCYRN1 forward—TAATCCCAG CTCTCAGGGAGGCTAA, BCYRN1 reverse—GGTTGTTGCTTTGAGGGAAGTTACGC, GAPDH-forward—ACCCACTCCTCCACCTTTG and GAPDH-reverse—CTCTTGTGCTCTTGCTGGG.

### Quadruplex prediction and experiments with synthetic RNAs

To identify probable quadruplex-forming regions within the PITX1 mRNA, the sequence (NCBI Accession # NM_002653.4) was analyzed using the QGRS Mapper software (34,35). Three regions with a G-score >40 were selected for further study. The corresponding synthetic RNAs were purchased from Integrated DNA Technologies with a 5′ Biotin tag and resuspended in 50 mM Tris, pH 7.6, and 100 mM KCl. The RNA sequences can be found in Figure 2A. Synthetic RNAs were confirmed by the supplier by mass spectrometry and provided desalted. RNA resuspension, quadruplex staining and electrophoretic mobility shift assays were performed as described previously (12,36). For electrophoretic mobility shift assays, RNA concentrations were kept constant at 150 nM, whereas protein was added in a concentration range from 0 to 750 nM to the binding reaction. Electrophoretic mobility shift assays with the full-length RHAU protein were resolved on 6% (w/v) polyacrylamide gels, whereas shifts with RHAU_53__–__105_ were resolved on 10% (w/v) polyacrylamide gels.

### Streptavidin pull-down assays

To assess RHAU interactions with the predicted quadruplex regions within the PITX1 mRNA, a streptavidin pull-down assay was performed with 5′-biotin-labelled PITX1 RNAs. Cell extraction was performed on 20 × 10^6^ cells per condition as described earlier in the text. A binding reaction was prepared that contained 500 µl of cell extract, 50 ng/µl Poly I-C, 250 µl of binding buffer (50 mM Tris-acetate, pH 7.8, 100 mM KCl, 10 mM NaCl, 3 mM MgCl_2_, 70 mM glycine, 10% (v/v) glycerol) and 250 nM biotin-labelled probe. Binding reactions were incubated for 30 min at room temperature, and 40 µl of Pierce streptavidin magnetic beads were subsequently added (Thermo-Fisher Scientific, Ottawa, ON, Canada). Following a 30-min incubation at room temperature, beads were washed three times in a 1:1 mixture of cytoplasmic and nuclear lysis buffers, followed by three washes each in cytoplasmic and nuclear lysis buffers and finally three washes in RIPA buffer [50 mM Tris, pH 8.0, 150 mM NaCl, 2 mM EDTA, 1% (v/v) nonidet P-40, 0.5% (w/v) sodium deoxycholate, 0.1% (w/v) sodium dodecyl sulphate (SDS)]. After washing, the beads were resuspended in 50 µl 1× SDS loading dye [62.6 mM Tris–HCl (pH 6.8), 2% (w/v) SDS, 0.01% (w/v) bromophenol blue, 10% (v/v) glycerol, 400 mM DTT], boiled for 5 min and the isolated proteins were separated by sodium dodecyl sulphate–polyacrylamide gel electrophoresis (SDS–PAGE) and assayed for binding by western blotting.

### siRNA, plasmid transfections and western blotting

The siRNA transfections were carried out using lipofectamine RNAiMAX (Invitrogen) using the reverse transfection protocol supplied by the manufacturer. For long-term siRNA knockdowns, cells were split and retransfected at 48 h post-transfection. Prevalidated siRNA duplexes were obtained from Invitrogen. The sequences of the siRNAs are as follows: DHX36—GGUGUUCGGAAAAUAGUAA (Cat. # s46823), PITX1—GCAACGUACGCACUUCACA (Cat. # s194685), DICER*—*GAUCCUAUGUUCAAUCUAA (Cat. # s23754) and AGO2—GGUCUAAAGGUGGAGAUAA (Cat. # s25931). As a control, all experiments were performed using an equal amount of the Medium GC StealthRNAi Universal Negative Control duplex (12 935-300) from Invitrogen Corporation (control siRNA). An siRNA-resistant silent mutant of the DHX36 cDNA was developed by site-directed mutagenesis of each codon within nucleotides 1713–1728 of the DHX36 mRNA (accession # NM_020865) from TCGGAAAATAGTAATT to GAGAAAGATTGTTATA in the pCDNA3 vector (Invitrogen). An ATPase-dead mutant of RHAU (RHAU E335A) was generated as previously published (37). An RSM-deficient mutant of RHAU (RHAU_ΔRSM_) was generated by deleting nucleotides 241–270 (NM_020865) corresponding to amino acids 54–63 (NP_065916) in a synthetic gene that comprised the 5′ region of the RHAU coding sequence through until an internal EcoRI restriction digest site. Plasmid transfections were performed using Turbofect (Thermo-Fisher Scientific) according to the manufacturer’s protocol. Western blotting was performed as described previously (12).

### Synthesis of the PITX1 3′ UTR, site-directed mutagenesis and β-galactosidase reporter assays

A 1071 nt sequence corresponding to the PITX1 3′ UTR was synthesized by Genscript Corporation (Piscataway NJ, USA). The PITX1 3′ UTR was cloned into a version of the PGL3-promoter vector (Promega Corporation, Madison WI, USA) in which the luciferase cDNA was replaced with cDNA encoding the β-galactosidase gene. The 3′ UTR was amplified by PCR using the following primers: B-gal-PITX1 UTR forward: GGCGGCGAATTCCAGCTGAGCGCCGGTCGCTACCATTACCAGTTGGT CTGGTGTCAAAAATAATAATAACCGCCCCGCCGCACCACGCGGGCCGGCGG and B-gal-PITX UTR reverse: ACGACGTCTAGATGCTTAGCACGCTCGGACTATGGTTTTAATAGACGTACATGGA and cloned using EcoRI and XbaI restriction enzyme sites. The 3′ UTR was cloned into the PGL3-promoter vector using an EcoRI site within the 3′ end of the β-galactosidase cDNA, allowing for direct fusion of the 3′ UTR following the β-galactosidase stop codon.

Quadruplex disrupting mutations were introduced into the reporter vector by site-directed mutagenesis with primers in which an internal run of guanines was mutated to cytosines. The following primers were used along with the appropriate corresponding antisense oligonucleotide: PITX1 Q1MUT sense: GGCCGGAGC GGGGAACCCCGCGGGCG, PITX1 Q2MUT sense: GATCCGTGTTGGGGCCCCCGTTGGGTTTGGGGG and PITX1 Q3MUT sense: AGCGGGCAGTGCCCCCCTGGCGGGAGG. Site-directed mutagenesis was performed using a previously published two single-primer reaction method (38). PITX1 1340–2110 and PITX1 2081–2383 were generated by standard PCR.

To performed reporter assays, cells were either left untransfected, or transfected with control or RHAU siRNA. After 48 h, cells were split 1:3 and retransfected. At 72 h cells were transfected with the β-galactosidase reporter vectors containing the PITX1 3′ UTR and various mutants. Lipofectamine RNAiMAX and lipofectamine LTX were used for the siRNA and cDNA transfections, respectively (Invitrogen). After an additional 24 h, cells were harvested and levels of β-galactosidase were assessed using the Promega β-galactosidase enzyme assay system (Promega Corporation). Absorbance measurements were made on a Biotech Epoch 96-well spectrophotometer (Fisher Scientific).

### RNase H digestion of bead-bound protein–RNA complexes

To identify the RHAU binding site on the PITX1 mRNA, DNA oligonucleotide-directed RNase H digestion of the protein-bound mRNA was performed. RNA immunoprecipitation was performed as described earlier in the text with a single confluent 150-mm dish of cells per RNase H digestion condition. Following binding of the protein–RNA complexes to the Protein A/G beads, the beads were washed once with each of the lysis buffer conditions described earlier in the text. Following washing, digestion reactions were prepared in a 100 µl volume that contained 10 µl of RNase H reaction buffer, 5 µM of each DNA oligonucleotide probe and 25 U of RNase H (New England Biolabs). Beads were resuspended in the digestion reaction and incubated for 45 min at 37°C with mixing every 10–15 min. Following digestion, the beads were washed once more with each buffer condition, and the remaining bound RNA was purified as described earlier in the text. A total of 5 µl of the purified RNA was amplified by quantitative PCR and compared with the undigested (RNase H without DNA oligonucleotide) sample. The RNA fragments that remained bound to the magnetic beads via RHAU interaction were detected by using primer sets within the PITX1 coding sequence (nucleotides 974–1143) and near the end of the 3′-UTR (nucleotides 2283–2376). All digestion reactions contained the same 5′ oligonucleotide cutting probe (the reverse complement of nucleotides 21–50 (PITX1_21__–__50_) of the mature mRNA (accession # NM_002653.4). The following DNA oligonucleotide probes were used to digest at the 3′-end: Set 1—PITX1_1311__–__1340_, Set 2—PITX1_1941__–__1970_, Set 3—PITX1_2081__–__2110_, Set 4—PITX1_2283__–__2303_ and Set 5—PITX1_2358__–__2376_.

## RESULTS

### RHAU interacts with the PITX1 mRNA

**Figure 1.**
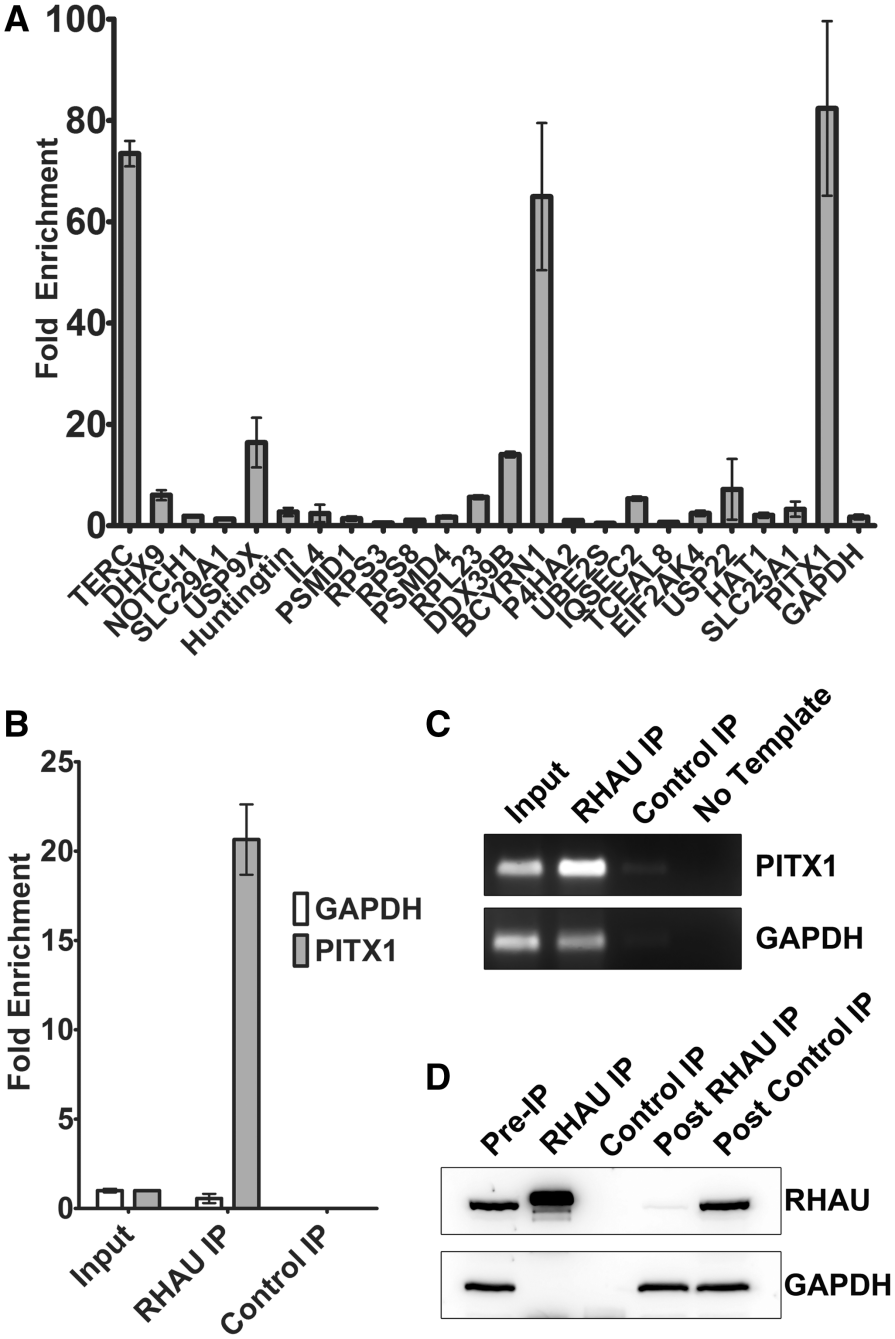
RHAU interacts with the PITX1 mRNA. (**A**) RT-PCR quantification of RHAU-RNA co-immunoprecipitations. Data represent the mean ± standard deviation. RHAU was immunoprecipitated from 5 mg of HEK293T cell lysates, and the co-precipitating RNA was used as a template for reverse transcription/real-time PCR with gene-specific primers. Fold enrichment values were calculated relative to an approximately equal (25 ng) quantity of total RNA. (**B**) Validation of PITX1 as a RHAU-interacting RNA. RHAU was immunoprecipitated from 500 µg of HEK293T cell lysate, and the co-precipitating RNA was purified as in (A). A total of 5 µl of the purified RNA was used as a template for reverse transcription/real-time PCR with primers specific for PITX1 and GAPDH. Fold enrichment values were calculated relative to an approximately equal (25 ng) quantity of total RNA. As a negative control, immunoprecipitations were performed in parallel with an isotype control antibody. RHAU immunoprecipitations resulted in a significant (∼20-fold) enrichment of the PITX1 mRNA but not the negative control (GAPDH mRNA). Data represent the mean ± standard error. (**C**) Amplification products were separated by agarose gel electrophoresis and stained with the SybrSafe DNA gel stain to ensure reaction specificity. (**D**) Western blot of HEK293T cell lysates before and following immunoprecipitation as well as 2% of the immunoprecipitation sample demonstrate specific and efficient immunoprecipitation of RHAU from cell lysates.

To identify novel RHAU interacting RNAs, a large-scale RHAU immunoprecipitation without cross-linking was carried out in both HEK293T and MCF-7 cells. Following immunoprecipitation, co-precipitating RNA was isolated, cloned and sequenced. A subset of the sequenced clones was selected for validation in a second RNA immunoprecipitation followed by specific detection by reverse transcription and quantitative real-time PCR (Figure 1A). Of this set, PITX1 was singled out for further study due to high and specific enrichment of the mRNA on RHAU immunoprecipitation. The interaction between RHAU and the PITX1 mRNA is demonstrated in Figure 1B, where an ∼20-fold enrichment of the PITX1 mRNA is observed in RHAU-precipitated RNA relative to an approximately equal amount (25 ng) of total RNA. This is in contrast to the GAPDH mRNA where we observe a relative decrease in abundance in the RHAU-precipitated RNA fraction. Furthermore, immunoprecipitation with an isotype control antibody [anti-synthetic hapten (Control IP)] demonstrates only trace amounts of both the PITX1 and GAPDH mRNAs. Specificity of the PCR reaction is confirmed by agarose gel electrophoresis of the reaction products (Figure 1C), and the efficiency of the RHAU immunoprecipitation is demonstrated by near complete RHAU depletion in the post-IP cell lysate (Figure 1D). Blots were reprobed with an anti-GAPDH antibody to demonstrate specificity of the IP. These results confirm a specific interaction between the endogenously expressed RHAU protein and the PITX1 mRNA.

### The PITX1 mRNA contains three quadruplex-forming sequences within the 3′-UTR that can interact with RHAU

**Figure 2.**
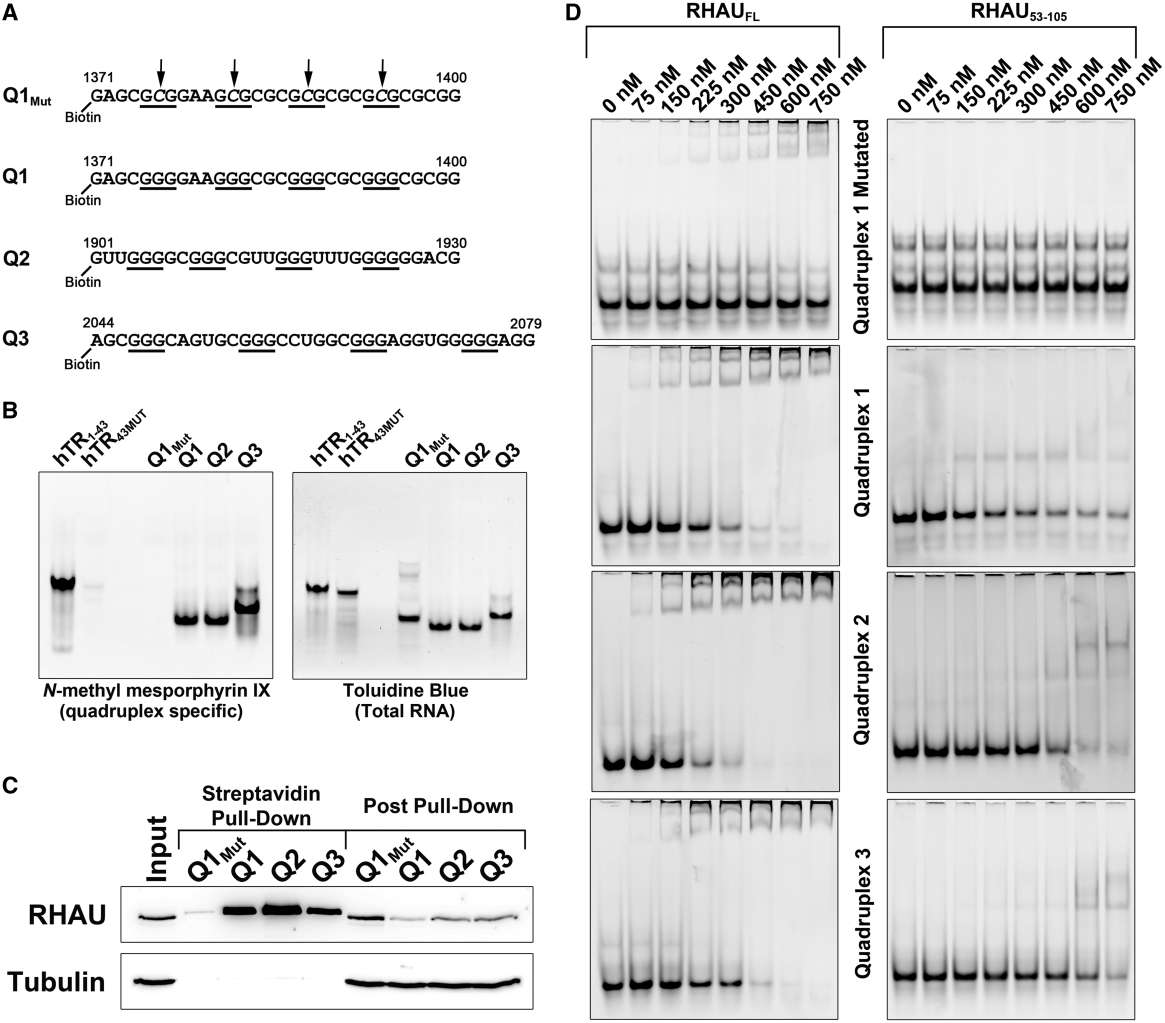
The PITX1 mRNA 3′-UTR contains three quadruplexes capable of interacting with RHAU. (**A**) Synthetic RNAs corresponding to quadruplex sequences identified in the PITX1 mRNA. Sequences are labelled Q1–3 corresponding to position along the mRNA. Position relative to the PITX1 mRNA is indicated at the 5′ and 3′ ends of each sequence, and guanine tracts are indicated by underscores. The Q1_Mut_ contains G-C substitutions in the middle of each guanine tract as indicated by arrows. (**B**) In all, 250 pmol of each RNA was separated by native-Tris-borate EDTA (TBE) polyacrylamide gel electrophoresis and stained with the quadruplex-specific dye *N*-methyl mesoporphyrin IX. Following fluorescent visualization, the gel was stained with the total RNA stain toluidine blue. Q1–3 demonstrates intense staining with *N*-methyl mesporphyrin IX, whereas the Q1_Mut_ RNA is undetectable. Two previously characterized RNAs (hTR_1–43_ and hTR_43MUT_) were included as positive and negative controls. (**C**) A streptavidin pull-down assay was performed with each of the PITX1-derived RNAs as well as the Q1_Mut_ RNA. Proteins bound to the biotinylated RNAs were detected by SDS–PAGE and western blotting. RHAU demonstrated strong affinity for each of the PITX1-derived quadruplex but not for the quadruplex-deficient control RNA (Q1_Mut_). (**D**) Electrophoretic mobility shift assays were performed with each of the previously described RNAs and the full-length RHAU protein as well as the RHAU_53–105_ truncation. RNA concentrations were maintained at 150 nM. Both RHAU and RHAU_53–105_ demonstrated specific binding to the quadruplex-forming RNAs but not the negative control RNA.

Previous studies have identified RHAU as a quadruplex-specific helicase with high affinity for both DNA and RNA inter- and intramolecular quadruplexes (12,13,39,40). Quadruplex interaction is mediated by an N-terminal region known as the RSM (14). An N-terminal fragment of RHAU (RHAU_53__–__105_) containing the RSM is sufficient to bind quadruplexes with both high specificity and affinity (12,14). To identify putative RHAU interacting regions within the PITX1 mRNA, the sequence was analyzed using QGRS Mapper (34,35). Three high scoring quadruplex regions were identified within the 3′-UTR and termed Q1–Q3. Synthetic RNAs corresponding to these sequences were synthesized with a 5′ biotin tag. As a negative control, a mutant of Q1 that contained G-C substitutions of the second nucleotide of each guanine tract was also synthesized (Figure 2A). Synthetic RNAs were analyzed by native polyacrylamide gel electrophoresis. Gels were stained with the quadruplex-specific fluorescent dye *N*-methyl mesoporphyrin IX (Figure 2B). As positive and negative controls, previously characterized quadruplex-forming and quadruplex-deficient RNAs derived from the human telomerase RNA were also analyzed (12). The putative quadruplex-forming sequences derived from the PITX1 mRNA each migrated primarily as a single species and demonstrated intense staining with *N*-methyl mesoporphyrin IX (Figure 2B). In contrast, the quadruplex-deficient mutant (Q1_Mut_) did not stain with the fluorescent dye. Following fluorescent visualization, the gel was subsequently stained with the total RNA stain toluidine blue to visualize the negative control RNAs.

To determine whether the PITX1-derived quadruplexes were capable of interacting with RHAU, the biotinylated RNAs were added to HEK293T cell lysates at a concentration of 250 nM. Biotinylated RNAs were recovered with streptavidin-conjugated magnetic beads, and proteins bound to the RNAs were separated by SDS–PAGE and identified by western blotting with specific antibodies. Each of the quadruplex-forming RNAs demonstrated strong enrichment of endogenous RHAU, whereas the quadruplex-deficient mutant showed only a faintly detectable quantity of bound RHAU (Figure 2C). To demonstrate a direct protein–RNA interaction, electrophoretic mobility shift assays were performed with each of the previously described RNAs and the purified full-length RHAU protein as well as an N-terminal truncation containing the RSM (RHAU_53__–__105_). Both the full-length protein and RHAU_53__–__105_ demonstrated a specific interaction with the quadruplex RNAs *in vitro*, indicating a direct interaction (Figure 2D). In agreement with previous studies, RHAU_53__–__105_ exhibited qualitatively lower affinity for the quadruplex RNAs as compared with the full-length protein (14). These data suggest that the PITX1 mRNA contains several regions within the 3′-UTR that are capable of folding into a G4-quadruplex and interacting with RHAU via the RSM.

### RHAU binds to the PITX1 3′-UTR between nucleotides 2110 and 2283

**Figure 3.**
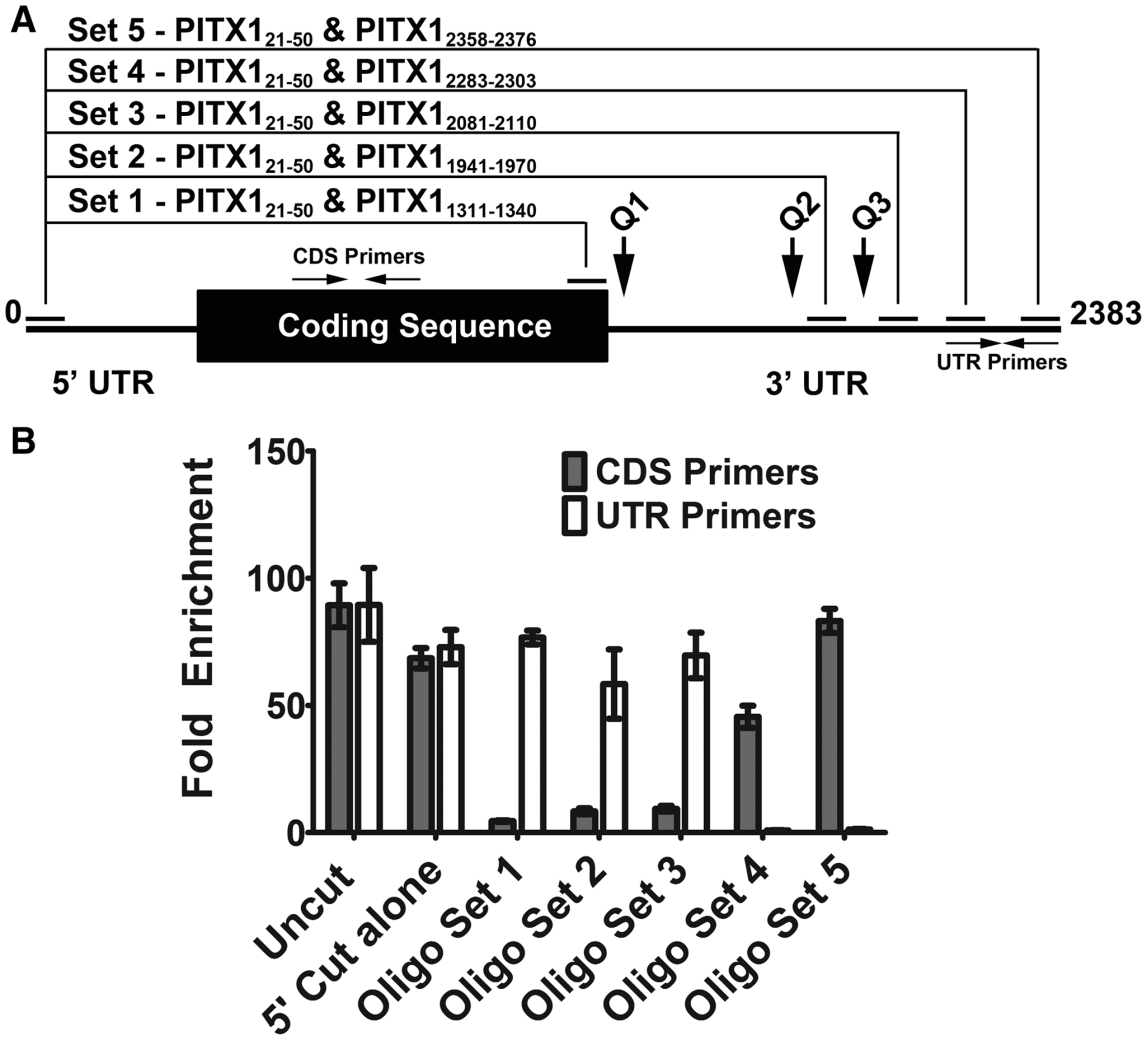
RHAU binds to the PITX1 3′-UTR between nucleotides 2110 and 2283. (**A**) Schematic representing the PITX1 mRNA. Primer binding sites are indicated by horizontal arrows, and DNA oligonucleotide complementary regions are indicated by solid horizontal lines. Relative locations of the predicted quadruplex-forming regions are indicated by vertical arrows. DNA oligonucleotide pairs used for RNase H-directed RNA digestion and their respective digestion sites on the full-length mRNA are indicated above. (**B**) RT-PCR analysis of bead-bound RNA following oligonucleotide-directed RNase digestion of the RNA–protein complex. Fold enrichment is expressed relative to 25 ng of total RNA for primer sets within the PITX1 coding sequence (CDS Primers) and 3′-UTR (UTR Primers). High fold enrichment is indicative of sustained binding of the primer template to RHAU following specific RNA digestion. Data represent the mean of three replicates ± standard error.

As the PITX1 mRNA contains three putative quadruplex-forming regions within the 3′-UTR that demonstrate efficient interaction with RHAU *in vitro*, specific digestion of the PITX1 mRNA in complex with endogenous RHAU protein was carried out to narrow down the *bona fide* interaction site. To perform this assay, endogenous RHAU was immunoprecipitated from HEK293T cell lysates. Following immunoprecipitation, DNA oligonucleotide pairs were used to direct RNase H-mediated digestion of the PITX1 mRNA (Figure 3A). Digestion reactions were performed on the mRNA while bound to Protein A/G beads via an antibody-RHAU linkage. Following digestion, beads were washed to remove the liberated RNA fragments, leaving only the portion of the mRNA bound to RHAU on the beads. The remaining RHAU-bound RNA was isolated and used as a template for reverse transcription and quantitative real-time PCR. By detecting the loss or retention of each primer template, we were able to narrow down the region of the mRNA bound by RHAU to the beads.

To detect the RHAU-bound RNA, primer sets were used within the PITX1 coding sequence (CDS Primers 974–1143) and near the end of the 3′-UTR (UTR Primers 2283–2376). All digestions shared a common 5′ targeting oligonucleotide complementary to nucleotides 21–50 of the PITX1 mRNA (PITX1_21__–__50_) to eliminate circularization of the mRNA. RNase H digestion with PITX1_21__–__50_ alone demonstrated a marginal reduction in the amplification with both the coding sequence (CDS) and UTR primer sets (Figure 3B). In contrast, digestion with PITX1_21__–__50_ and a DNA oligonucleotide immediately at the 3′ end of the PITX1 coding sequence (PITX1_1311__–__1340_) resulted in a loss of the CDS template, but the UTR template was retained via RHAU interaction on the beads. This result strongly suggests that the RHAU interaction exists within the 3′-UTR, as the 3′-UTR remains bound to RHAU when the 5′ UTR and coding sequence are dissociated by targeted digestion. Similar results were obtained using oligonucleotide sets that progressively truncated the mRNA from the 5′ end and removed all three putative quadruplex-forming regions of the PITX1 mRNA (Set 2—PITX1_1941__–__1970_, Set 3—PITX1_2081__–__2110_). Cutting further 5′ to this region with PITX1_2283__–__2303_ and PITX1_2358__–__2376_ resulted in ablation of the UTR primer binding sites and restoration of the amplification with the CDS primer set (Figure 3B). Retention of the CDS template on the beads indicates that the RHAU binding site resides between the two oligonucleotides used for digestion (Set 1 and 4). As digestion with Set 1 and Set 3 resulted in loss of the coding sequence template, it can be deduced that the binding site resides between the 5′ digestion oligonucleotides of Set 3 and 4 (PITX1_2081__–__2110_ and PITX1_2283__–__2303_). These data narrow the binding site of RHAU on the PITX1 mRNA to a ∼170 nt region that does not contain any of the predicted quadruplex-forming regions discussed previously; however, the results do not preclude the possibility that RHAU acts enzymatically on the identified quadruplexes while being retained at the mRNA via an as yet uncharacterized interaction.

### The endogenous PITX1 mRNA binds the RSM

**Figure 4.**
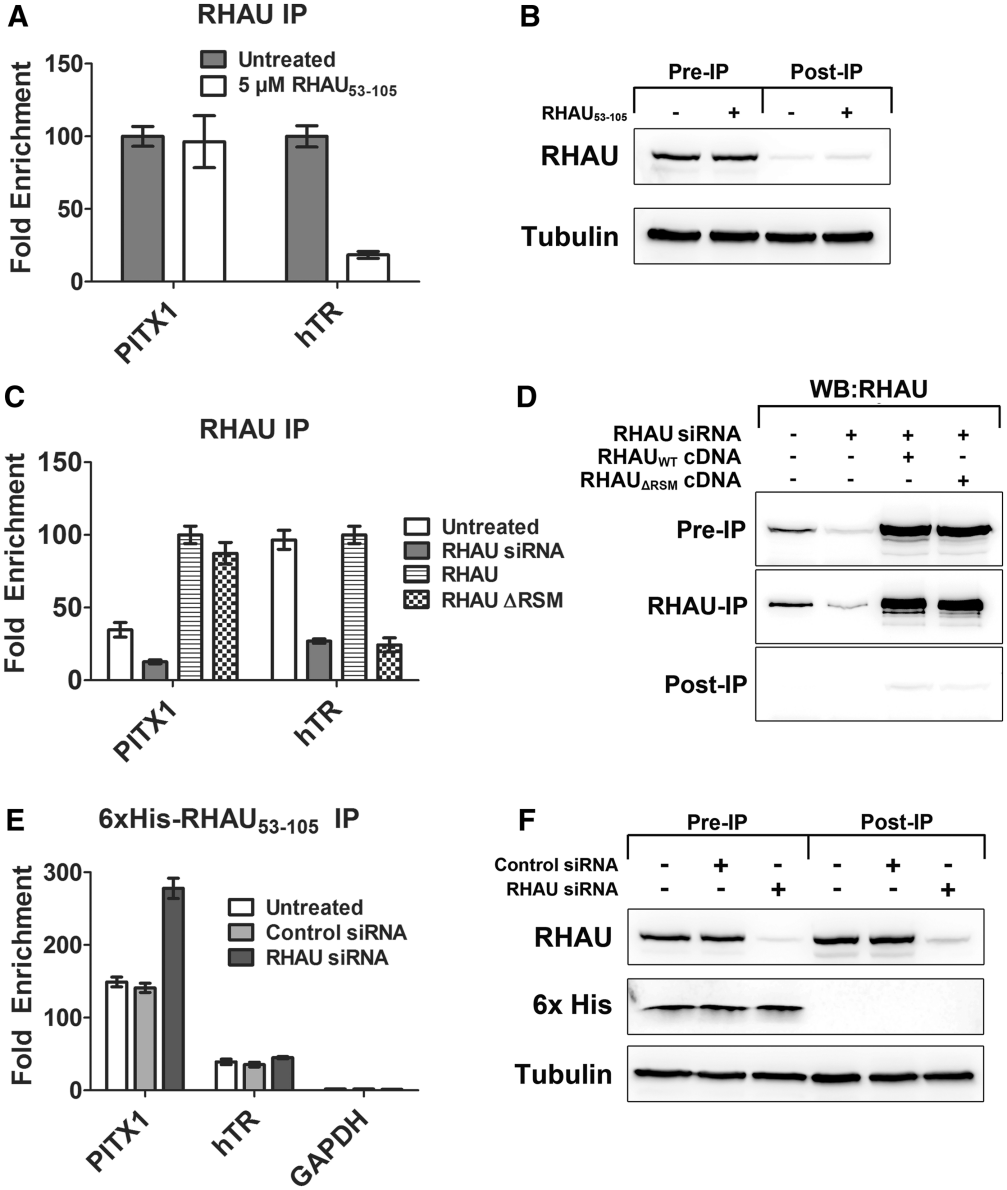
The endogenous PITX1 mRNA binds the RSM. (**A**) RNA immunoprecipitation experiment in which RHAU-RNA binding was competed with an excess of RHAU_53–105_ in the binding reaction. Fold enrichment values are expressed relative to the untreated sample (normalized to 100). Data represent the mean ± standard error. (**B**) Western blot demonstrating efficient RHAU immunoprecipitation in both the presence and absence of 5 µM RHAU_53–105_. (**C**) RNA immunoprecipitation experiment in which HEK293T cells were transfected with the wild-type RHAU cDNA and RHAU_ΔRSM_ cDNA. Following cDNA transfection, cells were transfected with RHAU siRNA to knockdown the endogenous RHAU protein. Enrichment of PITX1 and hTR were detected and compared with enrichment by the endogenous RHAU and in the context of siRNA knockdown alone. (**D**) Western blot demonstrating immunoprecipitation efficiency as well as resistance of cDNAs to siRNA knockdown. (**E**) HEK293T cell lysates were supplemented with 6×His-RHAU_53–105_, 6× was subsequently immunoprecipitated from the lysates, and co-precipitating RNA was purified and analyzed by RT-PCR with primers specific to PITX1, hTR and GAPDH. The 6×His-RHAU_53–105_ efficiently immunoprecipitated both the PITX1 and hTR RNA from HEK293T cell lysates, but did not demonstrate affinity for the negative control RNA GAPDH. The siRNA knockdown of RHAU resulted in a significant increase in PITX1 mRNA enrichment but had no impact on the other RNAs tested. Data represent the mean ± standard error. (**F**) Western blot demonstrating efficiency of RHAU knockdown by siRNA and the immunoprecipitation of 6×His-RHAU_53–105_.

As the PITX1 mRNA contained high-scoring quadruplexes that, when isolated, demonstrated quadruplex formation and specific RHAU interaction, but RNase H-mediated mapping of the RHAU-PITX1 mRNA interaction suggested binding that was independent of all three putative quadruplex-forming regions, we sought to further investigate quadruplex formation and the RHAU interaction in the context of the endogenous mRNA. To assess whether the interaction between RHAU and the PITX1 mRNA is mediated via the RSM (quadruplex interacting domain of RHAU), we performed a competition assay between the isolated RSM (RHAU_53__–__105_) and the endogenous RHAU protein. HEK293T cell lysates were supplemented with 5 µM RHAU_53__–__105_ for 30 min before RHAU immunoprecipitation. Co-precipitating RNAs were detected by quantitative real-time PCR. Primer sets were used for PITX1 as well as the telomerase RNA. The interaction between RHAU and human telomerase RNA is well characterized and consists of binding between the RSM and a quadruplex within the 5′ region of the RNA (12). Addition of an excess of RHAU_53__–__105_ had no impact on the interaction between RHAU and the PITX1 mRNA (Figure 4A). This is in stark contrast to the interaction with telomerase RNA, which exhibits a ∼5-fold reduction in enrichment by RHAU_53__–__105_ competition. These data support an interaction between RHAU and PITX1 that is independent of quadruplexes within the PITX1 mRNA and the RSM. Figure 4B demonstrates that the presence of an excess of RHAU_53__–__105_ has no impact on the immunoprecipitation efficiency of endogenous RHAU.

To further study the role of the quadruplex binding motif, an RSM-deficient RHAU cDNA was generated that also contained silent mutations within the siRNA targeting region of the mRNA. HEK293T cells were transfected with either the wild-type RHAU or the RHAU_ΔRSM_ cDNA. Following cDNA transfection, endogenous RHAU expression was knocked down by siRNA transfection for 48 h. Owing to silent mutations in the cDNA constructs, expression of both wild-type and RHAU_ΔRSM_ was not impacted by siRNA transfection (Figure 4D). After 48 h, a RHAU immunoprecipitation was performed on lysates from all samples. Figure 4C demonstrates that, as expected, RHAU knockdown by siRNA significantly reduced PITX1 and hTR RNA enrichment by RHAU IP. Reintroduction of the wild-type RHAU cDNA restored a strong enrichment of both the PITX1 and hTR RNAs by RHAU IP. RHAU_ΔRSM_ exhibited similar enrichment of PITX1 as the wild-type RHAU; however, enrichment of hTR was completely lost (similar to siRNA alone). These data confirm the findings of the competition assay performed in Figure 4A in that RHAU binds to the PITX1 mRNA in a manner independent of the RSM.

Although the competition assay (Figure 4A and B) and RHAUΔRSM experiments (Figure 4C and D) suggest that the interaction between RHAU and PITX1 is not quadruplex mediated, the PITX1 mRNA contains three putative quadruplexes (Figure 2) that are both capable of forming quadruplex and interacting with RHAU. To determine if quadruplexes exist within the endogenous PITX1 mRNA, we performed immunoprecipitations with a 6×His labelled version of the RSM. The 6×His-RHAU_53__–__105_ was added to HEK293T cell lysates at a concentration of 300 nM for 30 min. After this incubation, an equimolar amount of anti-6×His tag antibody was added to the binding reaction, followed by purification of protein–RNA complexes with Protein A/G magnetic beads. This experiment was performed with cells that were either left untreated or transfected with a control or RHAU-specific siRNA for 72 h. The 6×His-RHAU_53__–__105_ immunoprecipitation demonstrated strong enrichment of the PITX1 mRNA (∼150-fold) as well as the hTR mRNA (∼40-fold), whereas the GAPDH mRNA showed no significant enrichment (∼1.5-fold) (Figure 4E). RHAU knockdown by siRNA had no impact on the enrichment of hTR by 6×His-RHAU_53__–__105_ but resulted in a nearly 2-fold increase in the enrichment of the PITX1 mRNA (Figure 4E). RHAU knockdown and immunoprecipitation efficiency of 6×His-RHAU_53__–__105_ are demonstrated in Figure 4F. The 6×His-tagged recombinant protein retained the same quadruplex binding specificity and affinity as the untagged version of RHAU_53__–__105_ as determined by electrophoretic mobility shift assays (Supplementary Figure S1). Further supporting the hypothesis that RHAU is retained at the PITX1 mRNA in a quadruplex-independent manner, an siRNA-resistant and ATPase-deficient RHAU construct (RHAU E335A) did not demonstrate a significantly enhanced affinity for the PITX1 mRNA as compared with an siRNA-resistant wild-type RHAU cDNA when transfected into HEK293T cells in which endogenous RHAU had been knocked down by siRNA (Supplementary Figure S2).

### RHAU is a negative regulator of PITX1 protein expression

**Figure 5.**
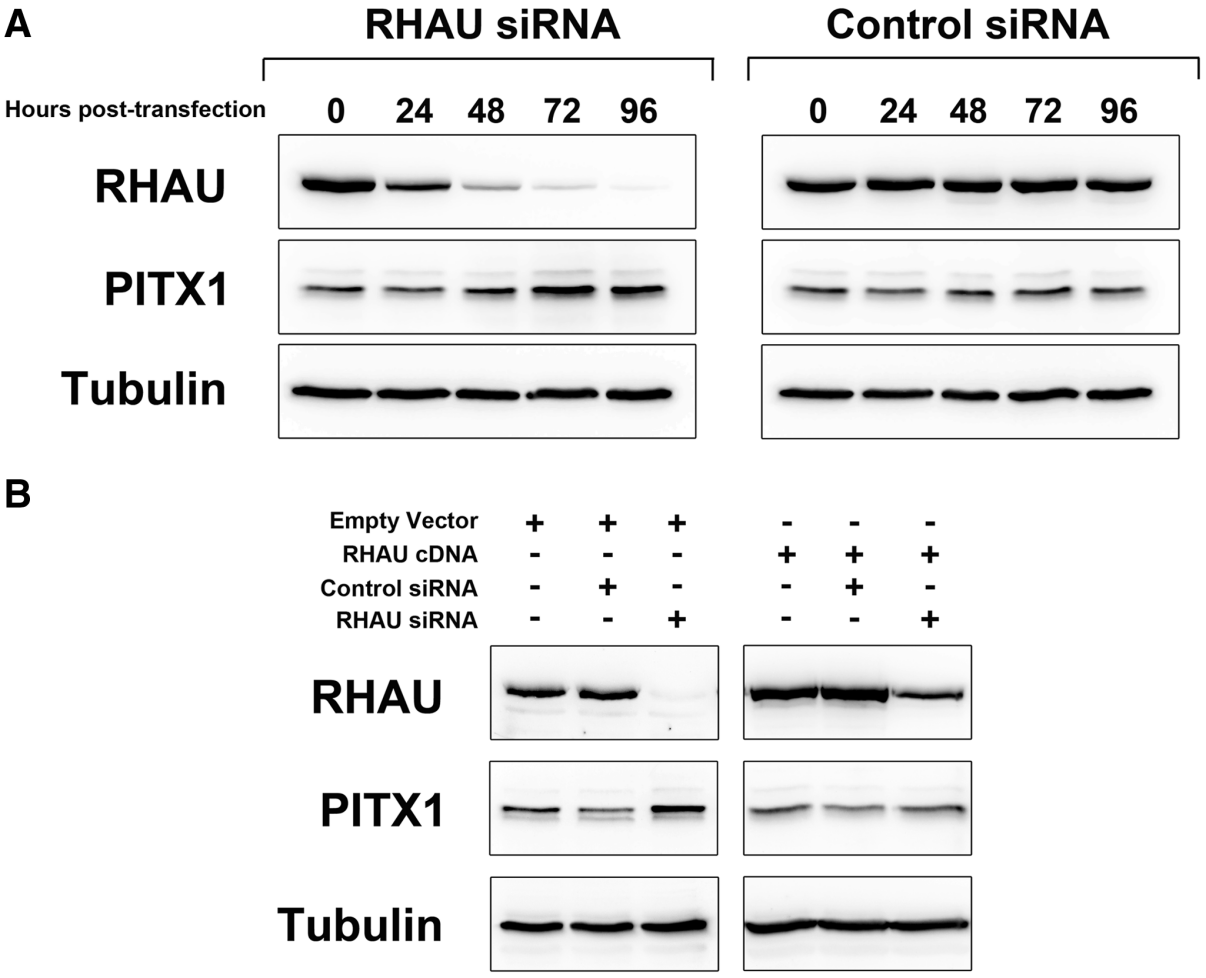
RHAU knockdown results in elevated expression of PITX1. (**A**) Western blot time-course analysis of PITX1 protein expression following transfection of RHAU and negative control siRNAs in HEK293T cells. Western blots were reprobed with RHAU antibodies to monitor RHAU knockdown efficiency as well as tubulin antibodies as a loading control. (**B**) The RHAU knockdown phenotype can be rescued by introduction of a plasmid containing silent mutations within the RHAU cDNA to confer siRNA resistance. RHAU expression persists following siRNA transfection in the RHAU cDNA transfected cells, and the elevation of PITX1 evident in the empty vector cells is abolished.

**Figure 6.**
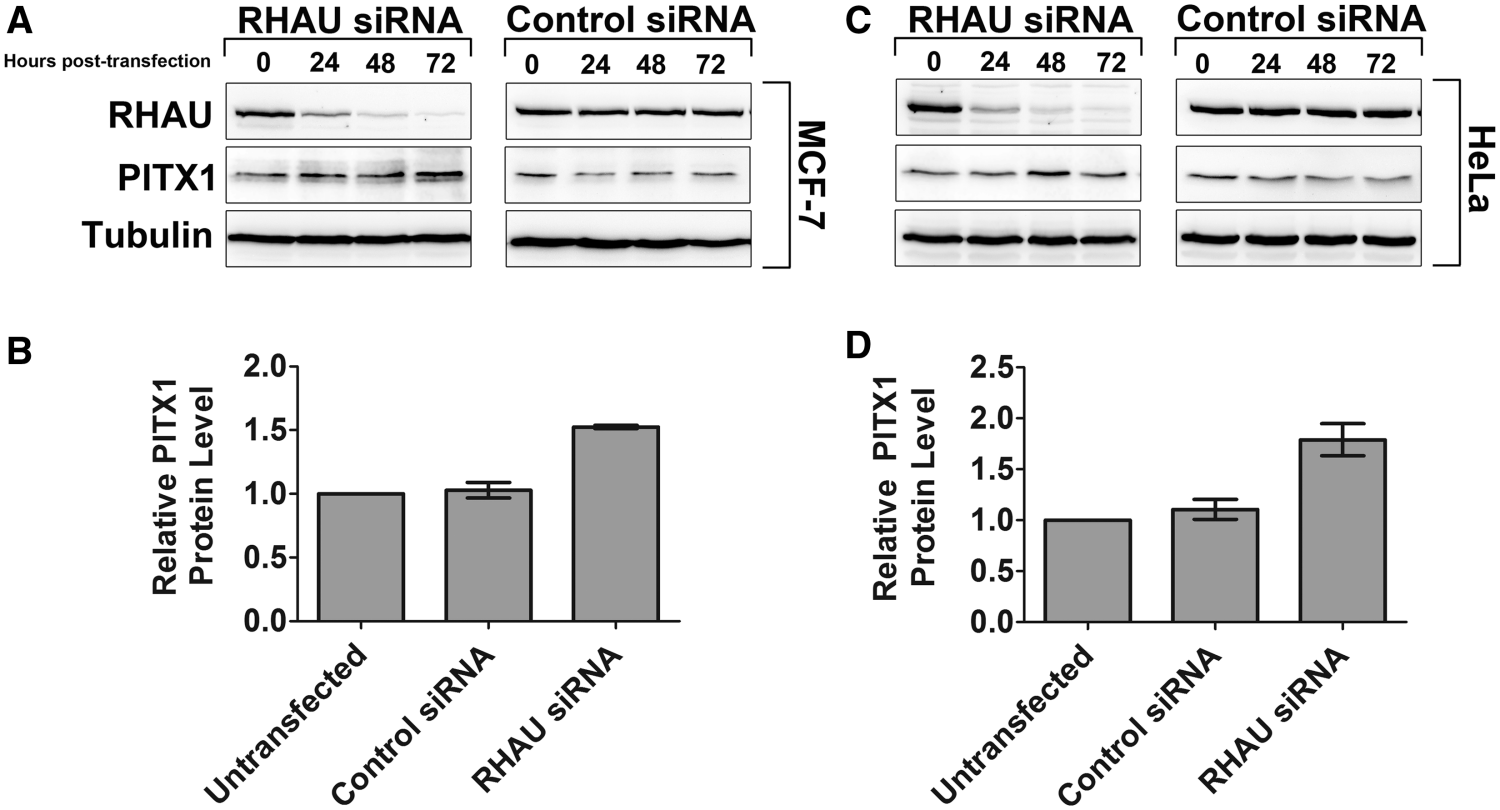
RHAU knockdown elevates protein expression in MCF-7 and HeLa cells. (**A**) Identical experiment as Figure 5 performed with MCF-7 cells for a 72 h time course. MCF-7 cells exhibit RHAU knockdown-dependent elevations in PITX1 protein expression within 72 h of siRNA transfection. (**B**) PITX1 levels in MCF-7 cells were quantified at the 72 h time point from three independent experiments. (**C**) Identical experiment as in (A) performed with HeLa cells. HeLa cells demonstrate an elevation in PITX1 protein expression by 48 h that returns to baseline at the 72 h time point. (**D**) PITX1 levels in HeLa cells were quantified at the 72 h time point from three independent experiments.

To investigate the functional consequences of the interaction between RHAU and the PITX1 mRNA, an siRNA time course was performed in HEK293T cells. Cells were transfected with siRNA specific to RHAU or a negative control siRNA for 96 h, and PITX1 protein levels were monitored every 24 h after transfection by western blot. Forty-eight hours following knockdown, an increase in PITX1 protein expression is evident that peaks at 72 h and persists through until the 96 h time point (Figure 5A). This is in contrast to the control siRNA samples in which no change in PITX1 protein level is evident. To assess RHAU knockdown efficiency, blots were reprobed with RHAU-specific antibodies as well as an anti-tubulin antibody as a loading control. To confirm that the observed increase in PITX1 protein expression was not due to off-target effects of the siRNA, a rescue experiment was performed by transfection of an siRNA-resistant mutant of the RHAU cDNA. This vector was developed by site-directed mutagenesis of each codon within the siRNA targeting site such that the protein coding sequence was unchanged and the siRNA targeting site was abolished. Cells were pretransfected with the RHAU cDNA or empty vector before transfection of the RHAU siRNA. Protein samples were analyzed at 72 h post-siRNA transfection, and western blotting was performed to detect RHAU and PITX1. As is demonstrated in Figure 5B, transfection of the siRNA-resistant RHAU cDNA prevented RHAU siRNA-mediated upregulation of PITX1. These data confirm that RHAU knockdown results in an increase in the expression level of PITX1 and suggest that RHAU acts as a negative regulator of PITX1 protein expression. Similar increases in PITX1 protein expression on RHAU knockdown were observed in both MCF-7 and HeLa cells (Figure 6, Supplementary Figure S3A and B).

### RHAU has a modest impact on PITX1 mRNA level

**Figure 7.**
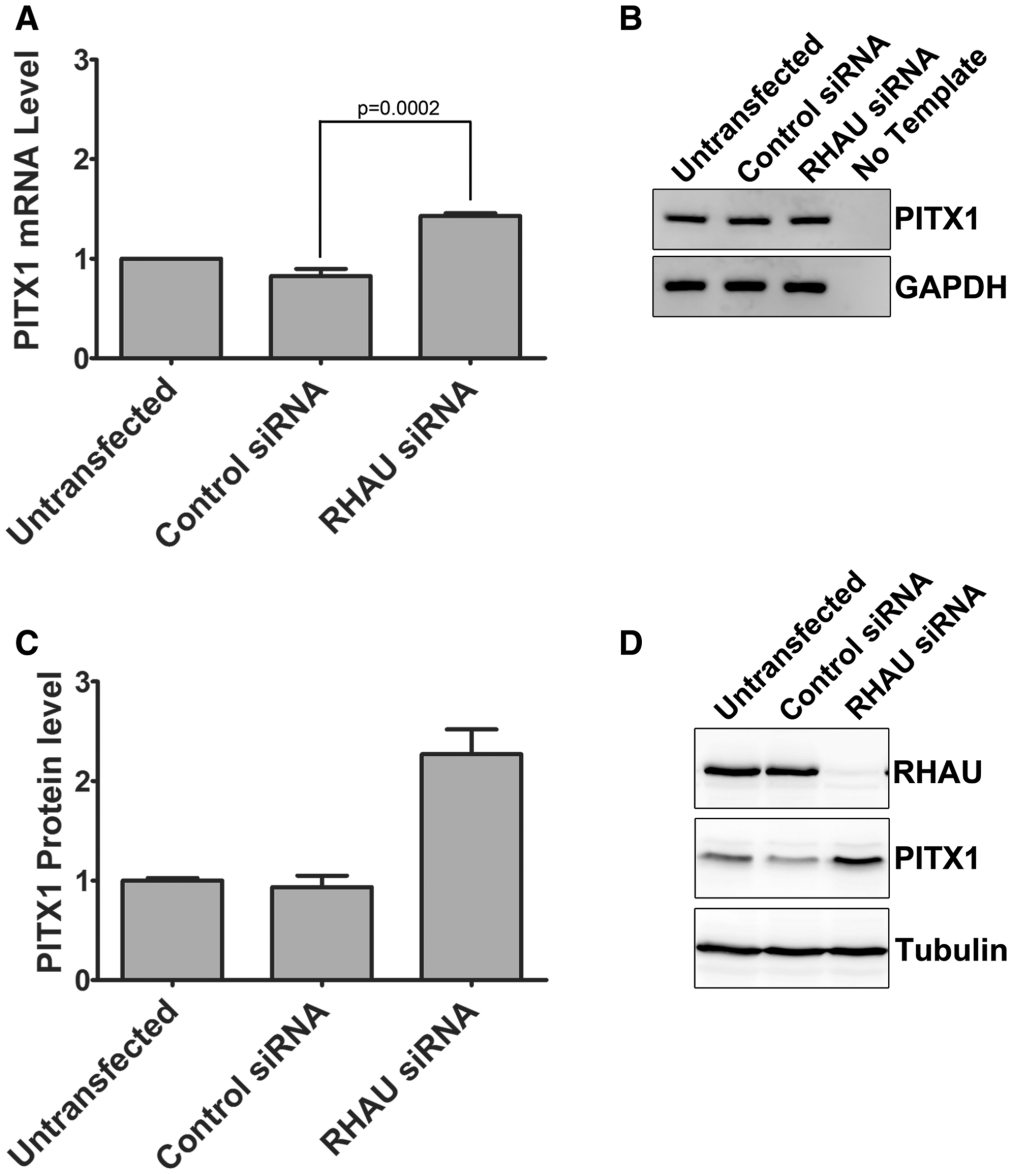
RHAU knockdown by siRNA has a modest impact on PITX1 mRNA level (**A**) HEK293T cells were transfected with RHAU or control siRNA for 72 h, and RNA was isolated and analyzed by RT-PCR. Data were normalized to the housekeeping gene GAPDH. RHAU knockdown by siRNA resulted in a ∼1.3-fold increase in the steady state level of the PITX1 mRNA as compared with untransfected cells. Data represent the mean of three replicates ± standard error. This increase was significant (*P* = 0.0002 by an unpaired 1 tail *t-*test). (**B**) Amplification products were separated by agarose gel electrophoresis and stained with the SybrSafe DNA gel stain to ensure reaction specificity. (**C**) RHAU knockdown by siRNA was performed three independent times followed by protein detection by western blot. Changes in PITX1 protein expression were quantified by densitometry and expressed relative to untransfected cells. (**D**) Representative western blot that provided the data for Figure 7C.

To assess whether the RHAU knockdown mediated elevation in PITX1 protein levels coincided with changes at the mRNA level, total RNA was isolated from HEK293T cells following 72 h knockdown with RHAU siRNA or control siRNA. PITX1 mRNA levels were assessed by quantitative real-time PCR and normalized to the housekeeping gene GAPDH. A modest (∼1.3-fold) yet significant (*P* = 0.0002, by unpaired, one-tailed *t*-test) increase in PITX1 mRNA levels is observed on RHAU knockdown by siRNA (Figure 7A). Amplification specificity was confirmed by agarose gel electrophoresis of the PCR reaction products (Figure 7B). Similar results were obtained using the PITX1 UTR primer set that amplifies a region immediately before the PITX1 polyadenylation site, indicating that RHAU knockdown does not shorten the 3′ UTR by altering the polyadenylation site of the PITX1 mRNA (data not shown).

To assess the change in PITX1 protein levels following RHAU knockdown, RHAU siRNA knockdown was performed in three independent experiments, and the change in PITX1 protein levels was measured by densitometry. Relative to the untransfected sample, PITX1 protein levels were elevated on average 2.3-fold following RHAU siRNA knockdown (Figure 7C and D). As the change in mRNA levels is considerably less, it is likely that the increases in PITX1 protein levels are due to an increase in translation of the PITX1 message, but could also be a combination of enhanced mRNA stability or increased transcription of the PITX1 gene.

### Quadruplexes within the PITX1 3′ UTR impact the basal expression and RHAU-dependent regulation of a β-galactosidase reporter construct

**Figure 8.**
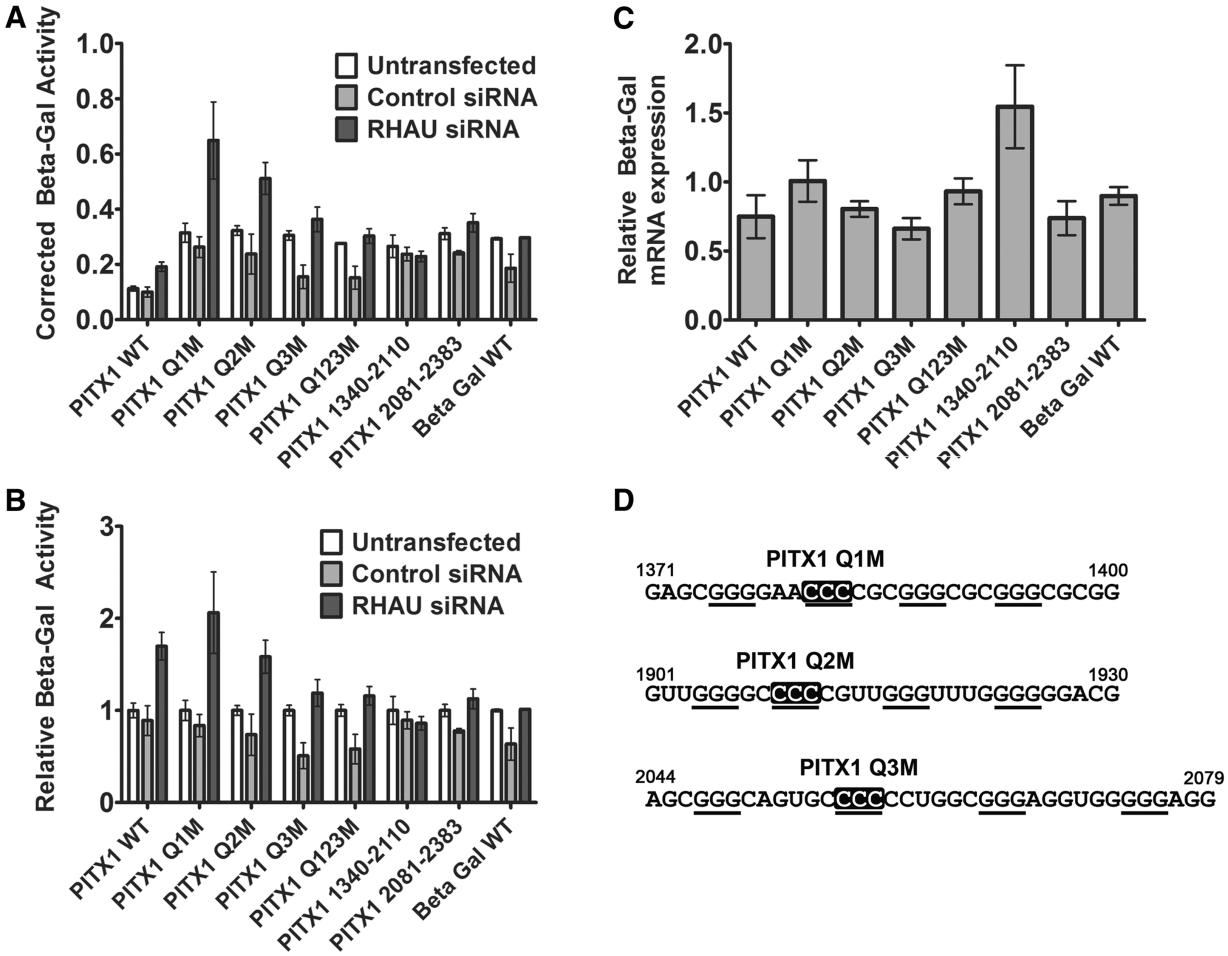
A β-galactosidase reporter construct containing the PITX1 3′ UTR exhibits similar RHAU-dependent expression regulation as endogenous PITX1. (**A**) The β-galactosidase assays were performed under conditions of control or RHAU siRNA transfection with the wild-type and quadruplex-deficient mutants of the PITX1 3′ UTR as well as two truncated UTRs. Absorbance values corrected for total protein concentration in the lysates are plotted for each condition. Data represent the mean of three experiments measured in duplicate ± standard error. (**B**) Identical data as in (A) normalized to the untransfected condition for each reporter construct. (**C**) The β-galactosidase mRNA levels in the non–siRNA-treated cells for each construct were monitored by RT-PCR and standardized to the housekeeping gene GAPDH. (**D**) Quadruplex-forming sequences from the PITX1 3′ UTR mutated in the reporter construct. Cytosines introduced in place of guanines are shown in white font with a black background.

To determine whether quadruplexes within the PITX1 3′ UTR play a role in the post-transcriptional regulation of the PITX1 mRNA, the PITX1 3′ UTR was cloned immediately downstream of the β-galactosidase cDNA. Quadruplex disrupting mutations were introduced into each of the three predicted quadruplex-forming regions by site-directed mutagenesis (Figure 8D). Mutations were introduced both individually (Q1M, Q2M and Q3M) and in combination (Q123M). The β-galactosidase cDNA without any additional 3′ sequence was used as an additional control. HEK293T cells were left untransfected or transfected with either the control or RHAU siRNA for before transfection of the β-galactosidase reporter plasmids. All of the reporter constructs containing the PITX1 3′ UTR demonstrate upregulation in the context of RHAU siRNA knockdown; however, this upregulation was reduced on disruption of quadruplex 3 (Q3M, Figure 8A and B) and in the triple mutant (Q123M). Data relative to the untransfected condition for each reporter are shown in Figure 8B to highlight RHAU siRNA-dependent upregulation of expression. No significant variation is observed in the total β-galactosidase mRNA level as measured by RT-PCR with primers specific to the β-galactosidase cDNA and standardized to the housekeeping gene Glyceraldehyde 3-phosphate dehydrogenase (GAPDH) (Figure 8C). Two additional constructs consisting of the UTR truncated immediately after the last predicted quadruplex (PITX1 1340–2110) and the UTR consisting solely of the sequence 3′ of the last predicted quadruplex (PITX1 2081–2383) were also tested. Both of these constructs demonstrated a reduced response to the RHAU siRNA, supporting the possibility that interplay between RHAU at the identified binding site and the upstream quadruplexes is necessary for the complete biological effect. Taken together, these data support a scenario in which RHAU is retained at the 3′ UTR independent of quadruplex interactions but that interactions with local quadruplexes play a role in RHAU-dependent regulation at the mRNA.

### Dicer knockdown increases PITX1 protein expression but does not impact RHAU association with the PITX1 mRNA

**Figure 9.**
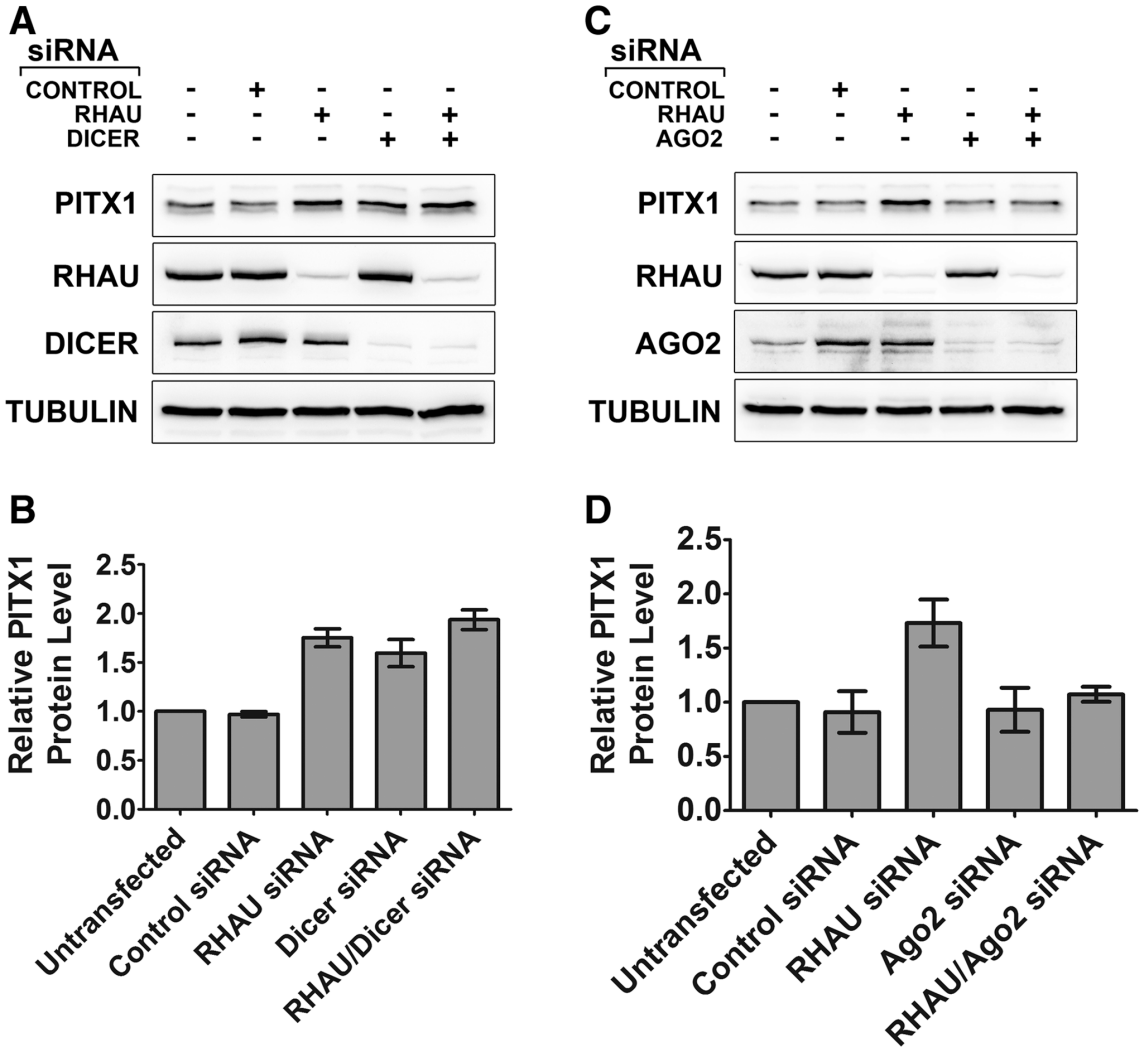
RHAU-mediated regulation of PITX1 protein expression involves components of the miRNA regulatory process. (**A**) PITX1 protein expression was monitored at 72 h following RHAU, Dicer and combined RHAU/Dicer knockdown by siRNA. Dicer knockdown by siRNA resulted in a similar increase in protein expression as compared with the RHAU knockdown. Combined knockdown of RHAU and Dicer was similar to knockdown of either protein alone. (**B**) Quantification of the PITX1 expression observed in (A) by densitometry. Additional gel images shown in Supplementary Figure S4. (**C**) PITX1 protein expression was monitored at 72 h following RHAU, Ago2 and combined RHAU/Ago2 knockdown by siRNA. Ago2 knockdown by siRNA had no observable impact on PITX1 protein expression. Combined RHAU/Ago2 knockdown resulted in a complete loss of RHAU siRNA-mediated upregulation of PITX1. (**D**) Quantification of the PITX1 expression observed in (B) by densitometry. Additional gel images shown in Supplementary Figure S4.

As RHAU binds to the 3′-UTR of the PITX1 mRNA, we sought to determine whether RHAU-dependent changes in PITX1 protein expression were related to miRNA-mediated regulatory mechanisms. To investigate miRNA-dependent regulation of PITX1, we performed siRNA knockdown of a key upstream miRNA processing enzyme, Dicer, a method previously demonstrated to significantly reduce levels of mature functional miRNAs (41). Dicer knockdown resulted in an elevation of PITX1 protein levels, comparable with that of RHAU knockdown (Figure 9A and B and Supplementary Figure S4A). Interestingly combined knockdown of RHAU and Dicer resulted in PITX1 protein levels that were similar to either knockdown alone, consistent with a shared mechanism of action. To investigate the possibility that RHAU associates with the PITX1 mRNA indirectly through interactions with an miRNA-dependent RNA-induced silencing complex (RISC), we assessed RHAU association with the PITX1 3′-UTR under conditions of either control siRNA or Dicer knockdown. Dicer knockdown did not significantly impact the interaction between RHAU and the PITX1 mRNA (Supplementary Figure S5A), and therefore the interaction appears to be independent of the presence of a Dicer processed miRNA.

### Ago2 expression is critical for RHAU knockdown-mediated increases in PITX1 protein expression

Small non-coding RNAs possess a myriad of functions within the cell, particularly within the context of post-transcriptional gene regulation (42). Mammalian argonaute proteins are important effector molecules in RNA-interference (RNAi)-based gene regulation. These proteins assemble with miRNAs and siRNAs to form a functional RISC that is directed to specific mRNAs via sequence complementarity with the bound guide RNA strand. RISC complexes participate in targeted RNA degradation as well as both translational repression and activation (42,43). Humans possess four argonaute family members (Ago1–4) of which Ago2 is the most abundantly expressed in HEK293T cells (44). For this reason, we sought to study the impact of Ago2 expression on PITX1. As Dicer knockdown resulted in upregulation of PITX1, we hypothesized that Ago2 knockdown should exhibit a similar impact. Furthermore, we wanted to test whether RHAU facilitates Ago2 interaction with the PITX1 3′-UTR. Transfection of control and RHAU siRNAs resulted in elevated expression of Ago2, a phenomenon reported previously (45). Unexpectedly, Ago2 knockdown had no observable impact on PITX1 expression and combined Ago2-RHAU knockdown abrogated RHAU knockdown-mediated elevation in PITX1 protein expression (Figure 9C and D and Supplementary Figure S4B). Furthermore, RHAU knockdown did not decrease but increased Ago2 association with the PITX1 mRNA (Supplementary Figure S5B). These data establish a link between RHAU and miRNA-mediated regulation of PITX1; however, further study is necessary to determine the precise interplay of factors that modulate translation of the PITX1 mRNA.

## DISCUSSION

In recent years, G4-quaduplexes have emerged from an *in vitro* obscurity to be recognized as multifunctional nucleic acid motifs capable of regulating key biological processes (8,46–48). Recent studies have solidified the *in vivo* presence and relevance of these structures for both multileveled gene regulation and maintenance of genomic integrity (2–4,7,49–50). Although RHAU was the initial helicase identified with quadruplex specificity, there is an ever-expanding catalogue of helicases and other proteins that possess quadruplex binding specificity and activity (50–53). As quadruplexes are capable of acting as molecular switches and lend themselves to the development of small molecule binding/stabilizing compounds, the potential for quadruplex-based therapeutic development is great (10–11,54–56). For this reason, expansion of our current knowledge of quadruplex-regulated biological processes will aid in defining novel molecular targets of quadruplex-based therapeutics as well as in understanding the biological implications of these compounds.

To identify novel targets of RHAU, we performed an RNA co-immunoprecipitation screen of the endogenously expressed protein from human cell lysates. Although a similar experiment was previously reported using a microarray-based analysis (16), the screen we performed has several advantages and will serve to complement the previous study. Although our methodology does not provide quantitative data, it does provide analysis of the total pool of precipitated RNAs allowing for identification of novel non-coding RNAs that may not be detected by the microarray assay. Additionally, unlike the previous report, our screen was performed with endogenous RHAU protein as opposed to an overexpressed tagged construct. To date, our screen has yielded ∼300 unique clones, and preliminary validation experiments identified the PITX1 mRNA as a specific and highly enriched RNA on RHAU immunoprecipitation. Although we have some observed overlap in the RNAs identified in our screen and those of the previously published results, we have identified many novel RNAs including PITX1. Validation and analysis of additional targets are currently underway, and we look forward to identifying new RHAU targets relevant to human health and disease.

Although RHAU has been demonstrated to bind non-quadruplex RNAs (19,20), several recent studies have bolstered its role as a quadruplex helicase; thus, multiple putative quadruplexes within the 3′-UTR of the PITX1 mRNA were the logical starting point in defining the interaction mechanism. Each of these regions within the PITX1 mRNA demonstrated an ability to fold into a quadruplex structure and interact with RHAU both in cell lysates and well-defined electrophoretic mobility shift assays. To analyze the interaction with the endogenous mRNA, we performed a unique RT-PCR-based variation of DNA oligonucleotide-directed RNase H-mediated RNA digestion. Although the traditional assay uses DNA oligonucleotides to identify regions of an RNA protected from RNase H digestion by a protein binding partner (57), we used DNA oligonucleotide probes to liberate progressively larger fragments of the PITX1 mRNA. These digestions were performed on the endogenous ribonucleoprotein complex that was immunoprecipitated from cell lysates and bound to magnetic beads via an antibody-RHAU linkage. This approach allowed for narrowing down the specific RHAU interaction site in an mRNA that is likely to have a multitude of RNA–protein contacts. This methodology may find future application in other ribonucleoprotein complex studies.

RNA fragments that were retained on the beads (and therefore bound by RHAU) were detected by RT-PCR with primer sets within the coding sequence and 3′-UTR. This approach identified the RHAU binding site as a ∼170 nt region near the 3′-end of the UTR that did not contain any of the putative quadruplexes. This assay could not further define the region, as additional DNA oligonucleotides within the region did not exhibit efficient RNase H-mediated digestion, likely due to secondary structure elements or bound protein that prevented hybridization. This region did, however, contain a predicted hsa-miR-19a binding site that was well conserved, raising the possibility that the RHAU interaction was mediated indirectly through RISC binding. However, preliminary studies of hsa-miR-19a using both an microRNA mimic and inhibitor did not yield significant changes in PITX1 expression (Supplementary Figure S6). Despite these negative data, an RISC-mediated interaction at the 3′ UTR warrants further investigation, as several reports have demonstrated an interaction between RHAU and mammalian argonaute proteins (18,21). Tethering of RHAU to the mRNA via this region does not rule out the possibility that RHAU interacts with and unwinds the quadruplexes within the 3′-UTR. This possibility is supported by β-galactosidase reporter assays that demonstrate that RHAU-dependent regulation of the PITX1 mRNA requires both quadruplex containing sequence of the 3′ UTR as well as the quadruplex-independent RHAU binding site. Mutations/truncations to the 3′ UTR that impacted both the quadruplexes and the RHAU interaction site independently altered RHAU knockdown-dependent expression changes. Clearly, further studies will be necessary to elucidate the mechanism by which RHAU is acting at the PITX1 mRNA. It is possible that RHAU modifications and/or other quadruplex binding proteins are at play in context of the mRNA making the precise mechanism complex. All reporter constructs also demonstrated repression in the context of the control siRNA transfection, a phenomenon that may be due to general enhancement of RNA interference pathways as is observed by increased expression of RISC components on siRNA transfection (Figure 9C).

The RSM is a well-defined RNA interaction motif within the RHAU protein that confers quadruplex binding specificity and does not exhibit affinity for single- or double-stranded RNA (12,14). To test whether the interaction between RHAU and the PITX1 mRNA is mediated by the RSM, we used an excess of the free RSM to compete with RHAU for RNA binding. These results demonstrated that the interaction between RHAU and PITX1 is distinct from the well-characterized interaction between RHAU and the telomerase RNA (12,16,17). To confirm these results, an RSM-deficient RHAU construct (RHAU_ΔRSM_) was tested and demonstrated similar affinity for the PITX1 mRNA as wild-type RHAU. These results indicate that the RSM is not solely mediating the interaction between the PITX1 mRNA and endogenous RHAU. Therefore, the interaction between RHAU and PITX1 is mediated via an alternative protein–RNA interaction domain in RHAU or is indirect and mediated via protein–protein interactions between RHAU and an unknown RNA binding protein. The precise mechanism of quadruplex-independent interaction of RHAU in this region is currently the subject of further study.

To further examine the putative quadruplex-forming regions within the context of the full-length mRNA, we performed RNA-immunoprecipitations with a 6×His-labelled recombinant RHAU truncation consisting solely of the minimal region that exhibits high and specific affinity for quadruplex RNA (RHAU_53__–__105_). The PITX1 mRNA was efficiently immunoprecipitated from HEK293T cell lysates with 6×His-RHAU_53__–__105_, as was the hTR RNA. This is in contrast to the negative control mRNA GAPDH that demonstrated no appreciable binding to RHAU_53__–__105_. 

Immunoprecipitation with the RSM demonstrated preferential enrichment of PITX1 (∼150-fold) as compared with hTR (∼40-fold), whereas immunoprecipitation of full-length RHAU demonstrates the opposite trend (∼20-fold enrichment of PITX1 and ∼100-fold enrichment of hTR)(12). This may be due to the presence of multiple quadruplexes within the PITX1 mRNA compared with a single quadruplex within hTR. Additionally, we observed an enhanced enrichment by 6×His-RHAU_53__–__105_ on RHAU knockdown by siRNA. This effect was not observed with the hTR RNA; however, we have previously demonstrated that a terminal quadruplex remains in the hTR RNA following remodelling of an internal quadruplex to double-stranded RNA (12). In light of this, it is not unexpected that enhanced enrichment is not observed with hTR.

As RHAU knockdown enhanced enrichment of the PITX1 mRNA by 6×His-RHAU_53__–__105_ despite that RHAU_53__–__105_ is not capable of displacing endogenous RHAU, it raises the possibility of increased quadruplex stability within the PITX1 mRNA in the absence of the helicase activity of RHAU. This supports a probable role for RHAU at the 3′-UTR as a quadruplex-specific helicase.

The results obtained demonstrate that the PITX1 mRNA contains quadruplex-forming sequences that are capable of interacting with the RSM both in cell lysates and in electrophoretic mobility shift assays. As the endogenous RHAU protein can bind to the mRNA in a quadruplex-independent manner, the possibility is raised that RHAU functions to unwind quadruplexes within the 3′-UTR even though they are not the principal mechanism by which the protein is bound. This would prevent dissociation of RHAU on quadruplex unwinding, allowing the mRNA to exist in a dynamic state with the helicase activity of RHAU remodelling the 3′-UTR to toggle between quadruplex and single/double-stranded RNA, thereby regulating binding of factors dependent on the particular fold that the RNA adopts.

Although the precise mechanism by which RHAU binds to and functions at the 3′-UTR of PITX1 has not yet been fully elucidated, the functional consequence of RHAU knockdown is an elevation of PITX1 protein levels. This result can be rescued by overexpression of an siRNA-resistant RHAU construct and is consistent across several cell lines tested. The elevation in protein levels in MCF-7 and HEK293T cells peaks at 72 h, whereas in HeLa cells the elevation occurs at 48 h and is returned to baseline by 72 h. This delayed response is likely due to the time between siRNA transfection and efficient decreases in the corresponding protein expression, a factor dependent on both the basal expression and half-life of the RHAU protein. The PITX1 mRNA steady-state level is only marginally increased on RHAU knockdown, suggesting the increases in PITX1 protein expression is primarily a result of enhanced message translation. Although previous studies have demonstrated RHAU-dependent changes in protein expression at the level of gene transcription and by modification of mRNA stability, this is the first study to support the possibility of RHAU-dependent translational regulation of an mRNA (5,6,20).

As the binding site for RHAU resides in the 3′-UTR of the PITX1 mRNA, and as a recent report demonstrated RHAU involvement in miRNA trafficking (19), we investigated whether RHAU-dependent changes in PITX1 expression were related to miRNA-based regulation of the PITX1 mRNA. Dicer knockdown resulted in a similar elevation of PITX1 protein expression as RHAU knockdown, and the combined knockdown of both RHAU and Dicer was non-additive. As we hypothesized RHAU may be bound to the mRNA through a bound RISC complex, we tested whether Dicer knockdown had any impact on RHAU association with the PITX1 mRNA. We found that Dicer knockdown did not impact RHAU association with the mRNA and next tested whether RHAU facilitated binding of the RISC to the mRNA. This would explain increases in PITX1 protein expression on RHAU knockdown, as the miRNA-mediated repression of PITX1 would be alleviated in the absence of RHAU expression. RHAU knockdown did not decrease Ago2 association with the PITX1 mRNA, unexpectedly Ago2 association was observed to increase under condition of RHAU knockdown. We also demonstrated that Ago2 knockdown abrogated RHAU siRNA-mediated PITX1 upregulation. This result is contrary to our initial expectations, but could be explained in part by the fact that Ago2 has been demonstrated to act also as a translational upregulator dependent on the context of the 3′UTR and bound cofactors (58,59).

The interplay between RHAU and miRNA machinery components appears complex and is currently the subject of further study. The data presented in this report establish a probable link between RHAU and RNA interference pathways, but our understanding of the mechanisms underlying this link remains preliminary. A recent report identified RHAU as a specific binding partner of the miR-134 microRNA and demonstrated a role in microRNA trafficking in neuronal cells (19). This raises two possibilities, first that the interaction between RHAU and the PITX1 mRNA is mediated by a microRNA and second that in certain contexts RHAU may be involved in the sub-cellular localization of the PITX1 mRNA.

This work identified a novel quadruplex-containing mRNA binding partner for the quadruplex-specific helicase RHAU. Unexpectedly, RHAU was discovered to bind independent of the quadruplex-forming regions of the mRNA and the quadruplex interacting motif of the protein. A direct consequence of RHAU knockdown by siRNA is observed in that PITX1 protein levels are elevated through translational regulation, mRNA stability or combination of the two factors. Although significant work lies ahead in defining the particular mechanism involved in both the recognition of the RNA by RHAU and the role RHAU plays in translational regulation, this study establishes a firm basis for future studies of the role of quadruplexes and quadruplex helicases within mRNA 3′-UTRs. Further studies of both PITX1 and other novel RHAU targets will paint a clearer picture of the particular RNA–protein and protein–protein interactions that facilitate the observed biological consequences of quadruplexes and their interacting proteins. Expansion of our knowledge of RHAU and quadruplex-mediated gene regulation will strengthen the platform for the development of quadruplex-targeted therapeutic strategies.

## SUPPLEMENTARY DATA

Supplementary Data are available at NAR Online.

## FUNDING

Canadian Insitutes of Health Research (CIHR)/Manitoba Health Research Council (MHRC) regional partnership program. MHRC post-doctoral fellowship (to E.B.). Canada Research Chair program (to J.S.). University of Manitoba undergraduate student research award (to R.H.). University of Manitoba Graduate Fellowship and Manitoba Graduate Scholarship (to S.D.). Faculty of Graduate Studies at the University of Manitoba (to E.D., O.M.). Funding for open access charge: Canadian Institutes of Health Research [RPA-118069].

*Conflict of interest statement*. None declared.

## Supplementary Material

Supplementary Data
