# Supplementary material for: The RNA helicase RHAU (DHX36) suppresses expression of the transcription factor PITX1
Source: Nucleic Acids Res. 2013 Dec 24;42(5):3346–61. doi: 10.1093/nar/gkt1340 (PMC3950718; doi:10.1093/nar/gkt1340)
Supplement: Supplementary Data [file supp_42_5_3346__index.html]

The RNA helicase RHAU (DHX36) suppresses expression of the transcription factor PITX1 — Supplementary Data 

# The RNA helicase RHAU (DHX36) suppresses expression of the transcription factor PITX1

## Supplementary Data

files

**Files in this Data Supplement:**

- Supplementary Data - pdf file
