# Supplementary material for: The RNA helicase RHAU (DHX36) suppresses expression of the transcription factor PITX1
Source: Nucleic Acids Res. 2013 Dec 24;42(5):3346–61. doi: 10.1093/nar/gkt1340 (PMC3950718; doi:10.1093/nar/gkt1340)

Supplementary Figure 1. **6xHis-RHAU$_{53-105}$ binds specifically to quadruplex RNA with similar affinity as untagged RHAU$_{53-105}$ but does not bind the control RNA. (A)** Electrophoretic mobility shift assay with 150 nM PITX1 quadruplex 1 RNA and increasing concentrations of 6xHis-RHAU$_{53-105}$. (B) Identical experiment as performed in (A) with the quadruplex-deficient mutant of PITX1 quadruplex 1.

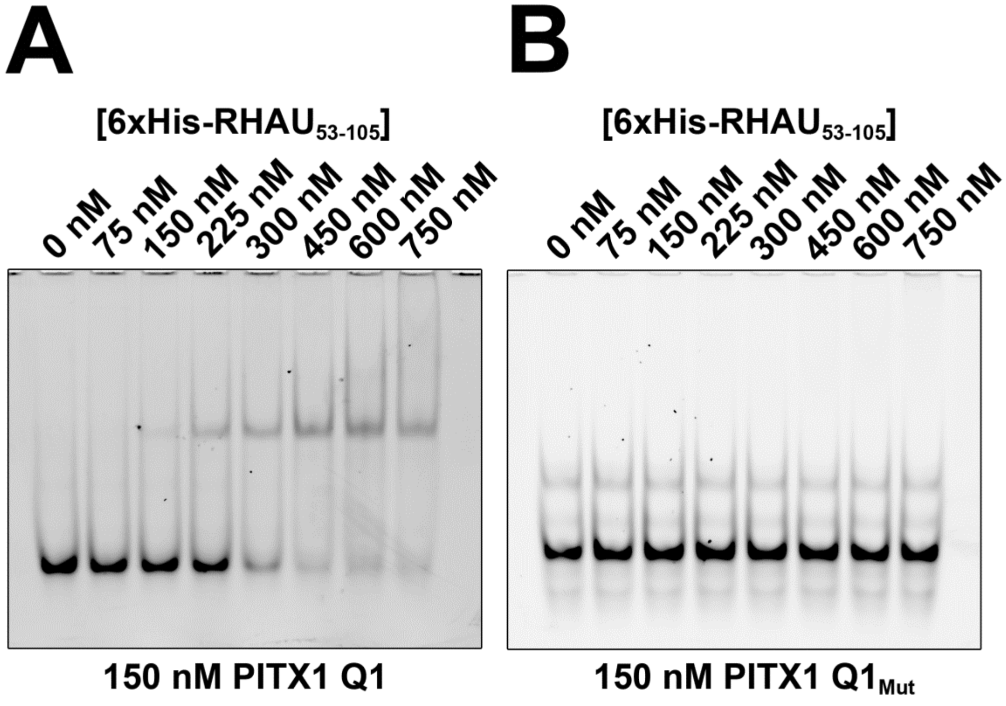

Supplementary Figure 2. **ATPase deficient RHAU (RHAU E335A) enriches the PITX1 mRNA to a similar extent as wild-type RHAU.** (A) HEK293T cells were untreated or transfected with RHAU siRNA followed by transfection of either an siRNA resistant wild-type RHAU cDNA or the siRNA resistant ATPase dead RHAU E335A cDNA. RNA immunoprecipitations were performed and co-precipitating PIX1 mRNA was assessed by RT-PCR. (B) Western blot confirming expression and immunodepletion of both endogenous and overexpressed RHAU.

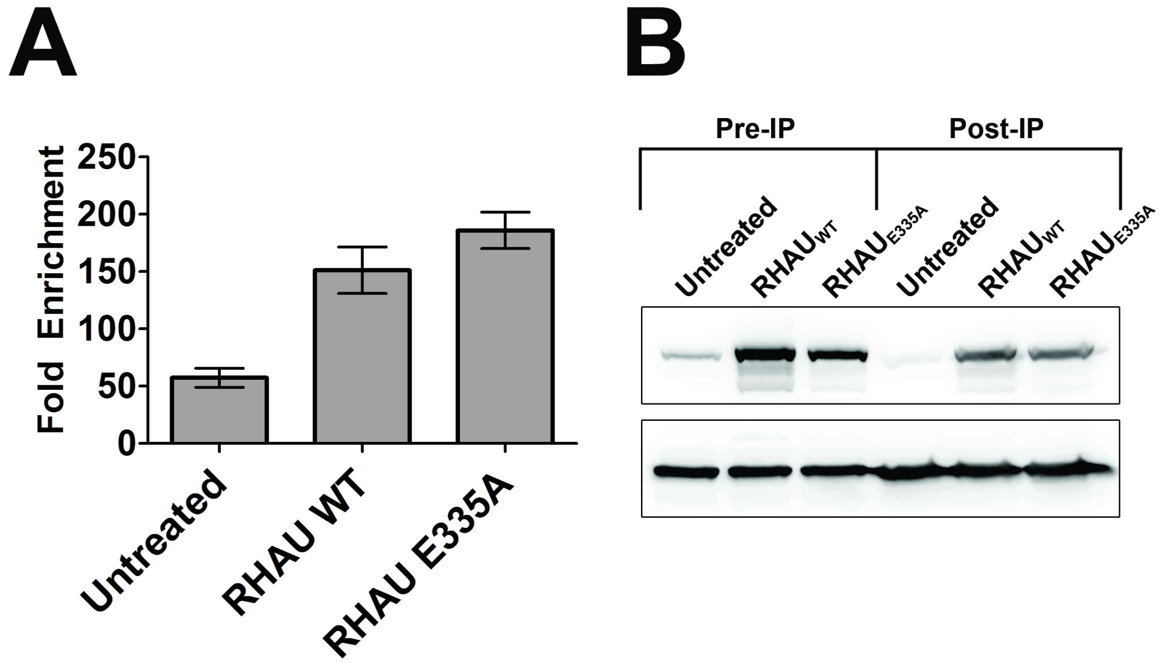

Supplementary Figure 3. **RHAU siRNA knock-down elevates PITX1 expression in HeLa and MCF-7 cells.** (A) Additional western blots of RHAU siRNA transfected HeLa cells that provide the quantification information used in Figure 6. (B) Additional western blots of RHAU siRNA transfected MCF-7 cells that provide the quantification information used in Figure 6.

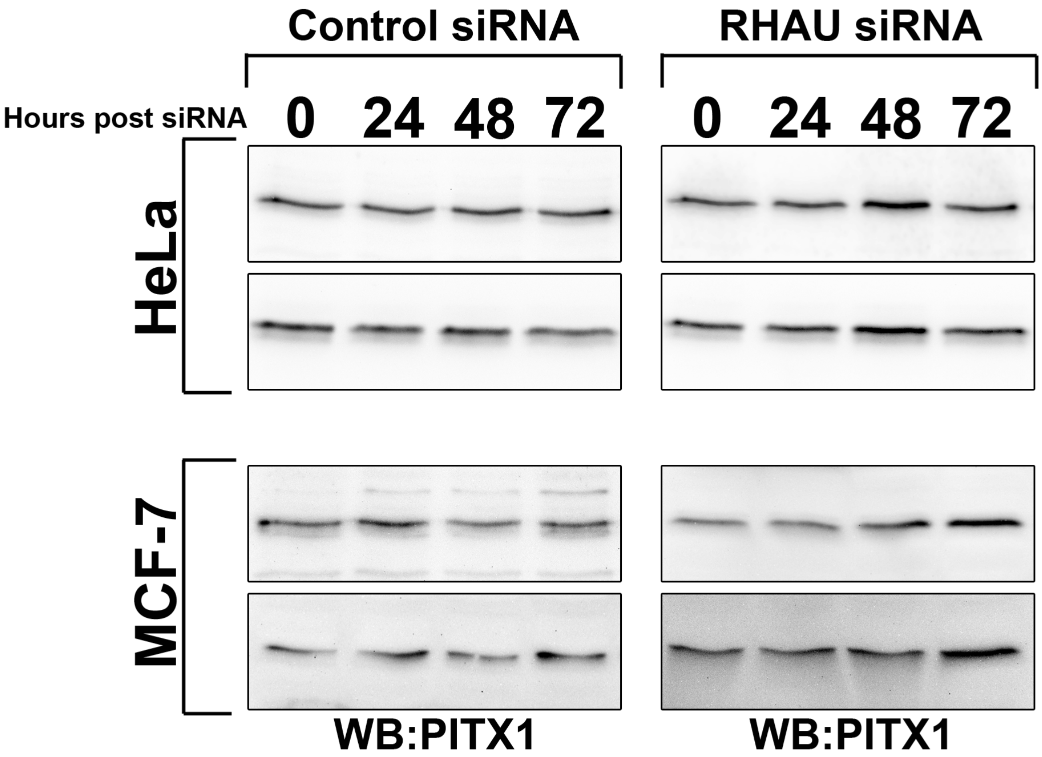

Supplementary Figure 4. **Western blot analysis of Dicer/RHAU and Ago2/RHAU siRNA knock-down experiments** (A),(B) Additional gel images that provide the quantification data found in Figure 9.

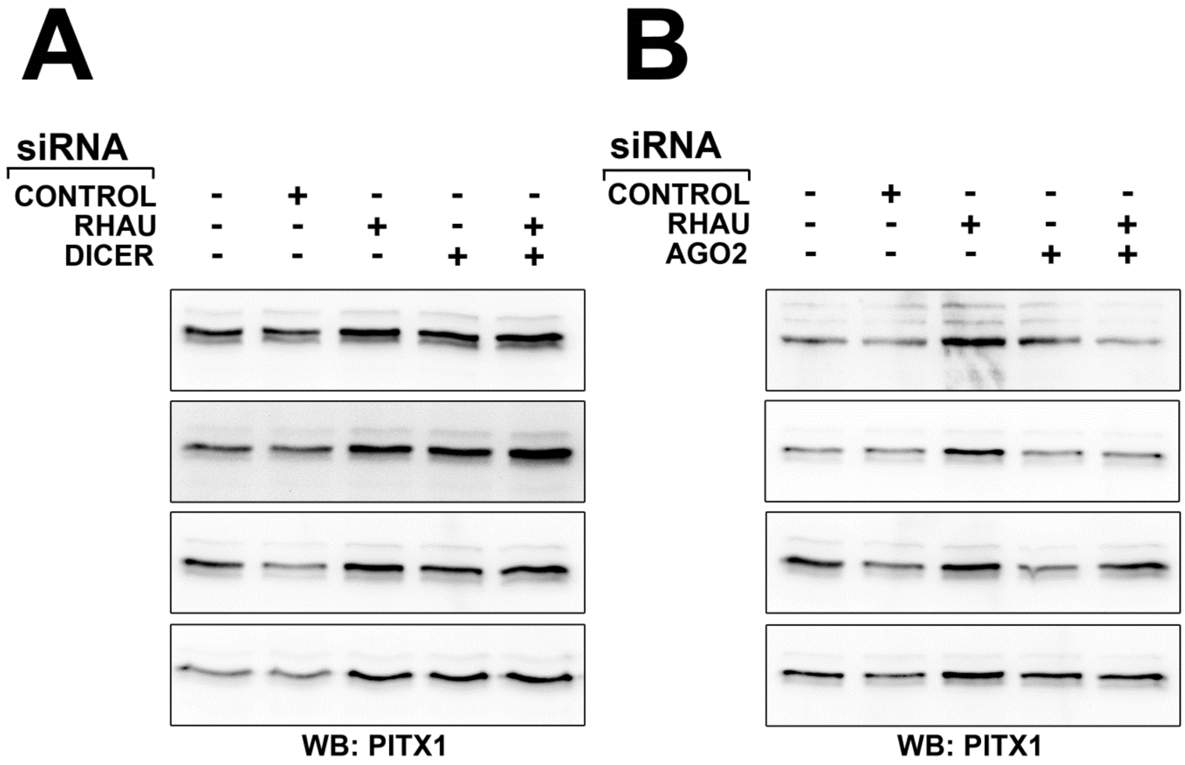

Supplementary Figure 5. **Impact of Dicer knock-down on RHAU association with the PITX1 mRNA and impact of RHAU knock-down on Argonaute 2 binding to the PITX1 mRNA.** (A) RNA immunoprecipitation experiment in which RHAU was immunoprecipitated from HEK293T cells transfected with either control or Dicer siRNA. Data represents mean enrichment +/- standard error. (B) RNA immunoprecipitation experiment in which Ago2 was immunoprecipitated from HEK293T cells transfected with either control or RHAU siRNA. Data represents mean enrichment +/- standard error.

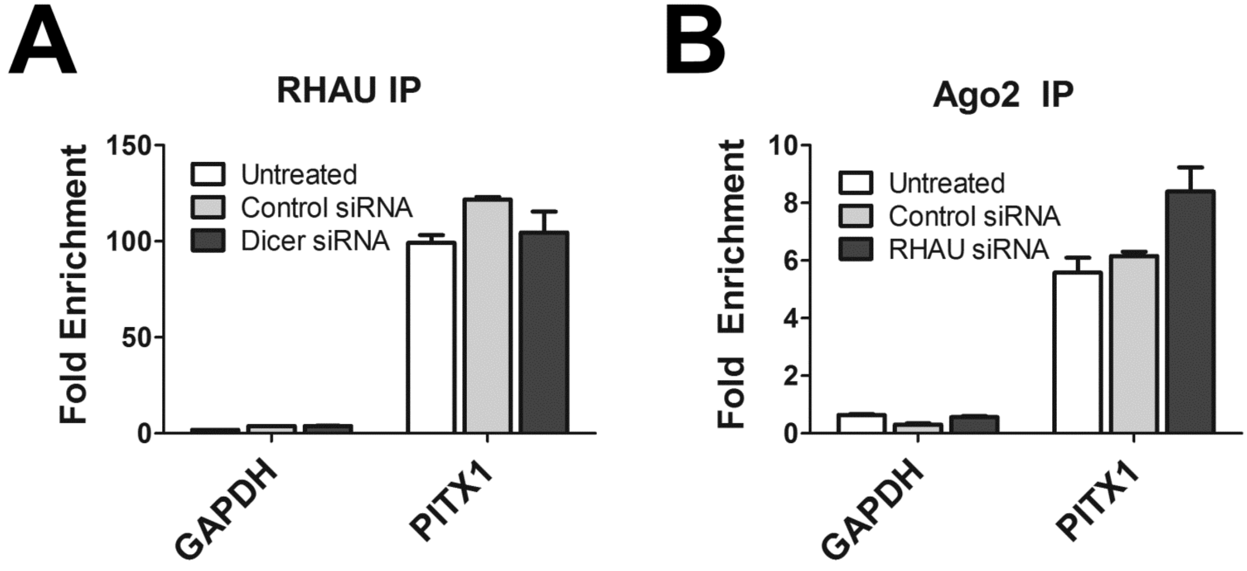

Supplementary Figure 6. **hsa-miR19a does not impact PITX1 protein expression in HEK293T cells.** (A) HEK293T cells were transfected with 50 pmoles of either a control siRNA, an hsa-miR19a mimic (MISSION microRNA mimic,HMI0344, Sigma-Aldrich) or an hsa-miR19a anti-sense inhibitor (mercury LNA Power Inhibitor, 426920-00, EXIQON). 48 hours post-transfection cells were lysed and total protein analyzed by SDS-PAGE and western blotting. Blots were probed with anti-PITX1 antibodies as well as an anti-Tubulin antibody as a loading control.

A

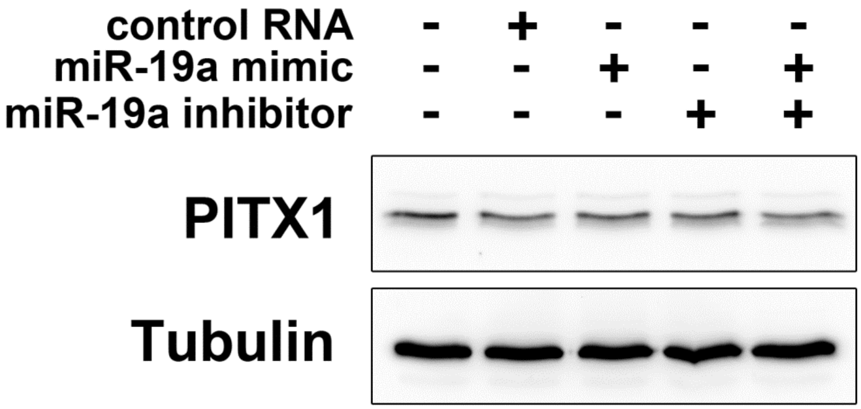

Supplement: Supplementary Data [file supp_gkt1340_nar-01570-f-2013-File011.pdf]
